# Protein kinase TgCDPK7 regulates vesicular trafficking and phospholipid synthesis in *Toxoplasma gondii*

**DOI:** 10.1371/journal.ppat.1009325

**Published:** 2021-02-26

**Authors:** Priyanka Bansal, Neelam Antil, Manish Kumar, Yoshiki Yamaryo-Botté, Rahul Singh Rawat, Sneha Pinto, Keshava K. Datta, Nicholas J. Katris, Cyrille Y. Botté, T. S. Keshava Prasad, Pushkar Sharma

**Affiliations:** 1 Eukaryotic Gene Expression laboratory, National Institute of Immunology, New Delhi, India; 2 Institute of Bioinformatics, International Tech Park, Bangalore, India; 3 Amrita School of Biotechnology, Amrita Vishwa Vidyapeetham, Kollam, India; 4 ApicoLipid Team, Institute of Advanced Biosciences, CNRS UMR5309, INSERM U1209, Université Grenoble Alpes, Grenoble, France; 5 Center for Systems Biology and Molecular Medicine, Yenepoya Research Centre, Yenepoya (Deemed to be University), Mangalore, India; 6 NIMHANS IOB Proteomics and Bioinformatics Laboratory, Neurobiology Research Centre, National Institute of Mental Health and Neuro Sciences, Bangalore, Karnataka, India; University of South Florida, UNITED STATES

## Abstract

Apicomplexan parasites are causative agents of major human diseases. Calcium Dependent Protein Kinases (CDPKs) are crucial components for the intracellular development of apicomplexan parasites and are thus considered attractive drug targets. CDPK7 is an atypical member of this family, which initial characterization suggested to be critical for intracellular development of both *Apicomplexa Plasmodium falciparum* and *Toxoplasma gondii*. However, the mechanisms via which it regulates parasite replication have remained unknown. We performed quantitative phosphoproteomics of *T*. *gondii* lacking TgCDPK7 to identify its parasitic targets. Our analysis lead to the identification of several putative TgCDPK7 substrates implicated in critical processes like phospholipid (PL) synthesis and vesicular trafficking. Strikingly, phosphorylation of TgRab11a via TgCDPK7 was critical for parasite intracellular development and protein trafficking. Lipidomic analysis combined with biochemical and cellular studies confirmed that TgCDPK7 regulates phosphatidylethanolamine (PE) levels in *T*. *gondii*. These studies provide novel insights into the regulation of these processes that are critical for parasite development by TgCDPK7.

## Author summary

In this study, we demonstrate that protein kinase TgCDPK7 regulates cellular processes like vesicular trafficking and the synthesis of phospholipids, which are critical for the development of the parasite *Toxoplasma gondii*. It regulates the localization of a small GTPase TgRab11a by phosphorylating it at a specific site, which is critical for trafficking of important parasite proteins and is important for parasite division. TgCDPK7 may regulate key enzymes involved biogenesis of phosphatidylethanolamine, which may contribute to the synthesis of this important phospholipid. These and other studies shed light on a novel signaling pathway in apicomplexan parasite *Toxoplasma gondii*.

## Introduction

Apicomplexan parasite *Toxoplasma gondii* is an obligate intracellular protozoan, which has to invade host cells to proliferate and thus survive. The lytic cycle of *T*.*gondii* causes the acute form of the disease through the rapid division of tachyzoites via the process of endodyogeny, which involves the formation of two daughter cells within the mother cell. Tachyzoites replicate within almost any nucleated cell from a warm-blooded host by generation cycles of 6 to 8h (*in vitro*), until they reach a number of 64 to 128 parasites per infected cell. They can then trigger active egress, which involves lipid signal transduction [1] and are then able to invade neighboring cells. There are several processes that *Toxoplasma* shares with related apicomplexan *Plasmodium*, which causes malaria. Therefore, understanding molecular mechanisms that regulate their development will unravel novel insights into their biology and also aid development of novel strategies against diseases like Toxoplasmosis and malaria.

**Fig 1:**
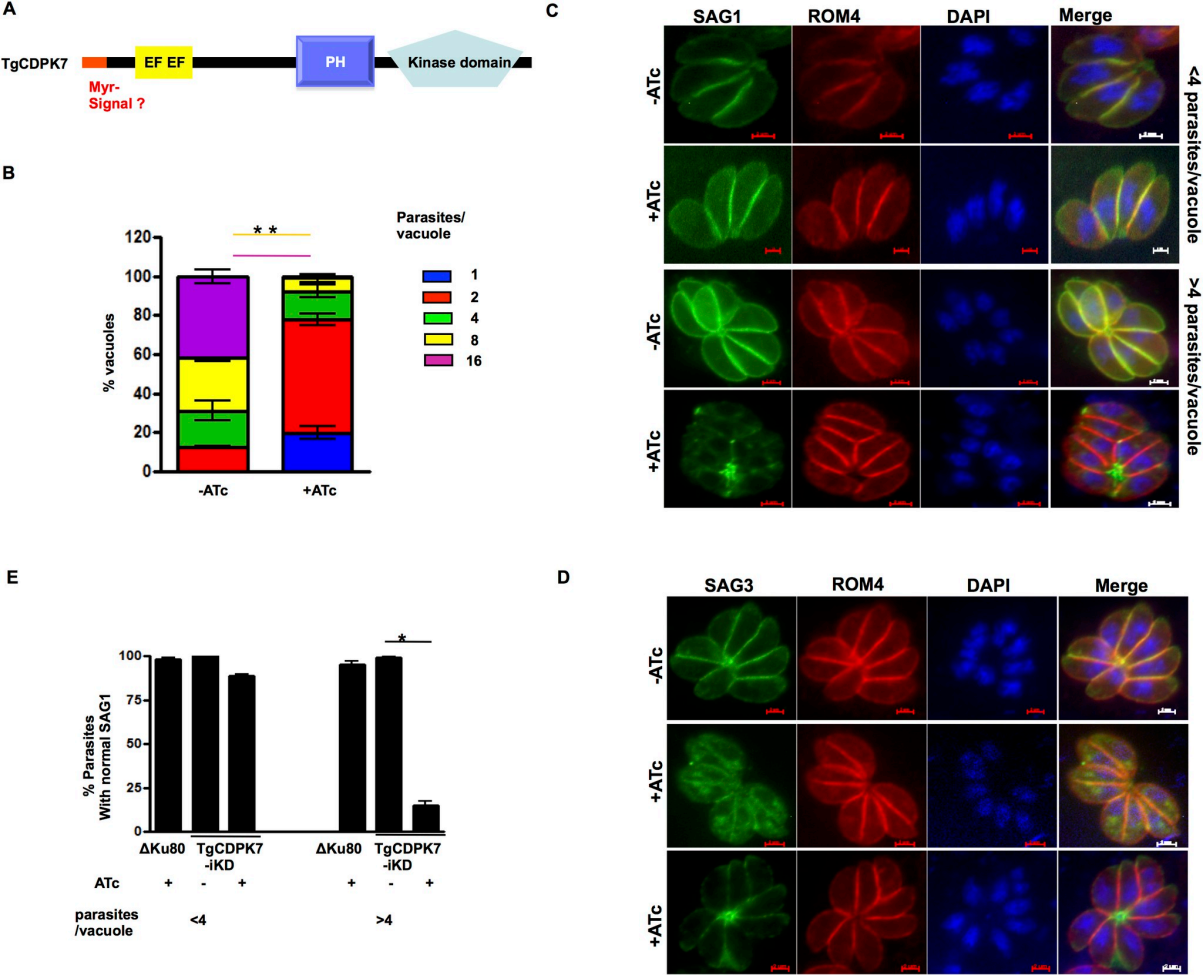
TgCDPK7 is critical for parasite division and localization of SAG1/3. **A.** Schematic representation of TgCDPK7. It contains a PH domain adjacent to the kinase domain at the C-terminus and two EF hand motifs near the N-terminus. In addition, the TgCDPK7 contains a putative myristoylation motif (red) near its N-terminus. **B.** TgCDPK7-iKD parasites were pre-incubated for 48h with ATc and were subsequently allowed to invade fresh HFFs in the presence or absence of ATc. The number of parasites per vacuole was determined after 24h. Data represent mean ± SE, n = 3 and at least 200 vacuoles were counted for each condition (n = 3*, p<0.001 for 8/16 parasites/vacuole, ANOVA). **C and D.** TgCDPK7-iKD or ΔKu80 parasites were treated with 1μg/ml ATc for 2 days. After parasites egressed, fresh HFF were infected along with additional 24h ATc treatment and IFA was performed on untreated or ATc treated parasites using SAG1 (C) or SAG3 (D) and ROM4 antibodies. **E.** Quantification of vacuoles containing normal SAG1 staining, which was at parasite periphery, from experiments described in panel C. Data are mean ± SE of three independent experiments and at least 200 vacuoles were counted for each condition (*, n = 4, p<0.001, ANOVA).

Calcium Dependent Protein Kinases (CDPKs) are major effectors of calcium signaling in plants, protists and apicomplexan parasites. They regulate key parasitic processes in *Plasmodium* and *Toxoplasma* like host cell invasion, egress and sexual differentiation [2–11]. Typically, CDPKs contain a S/T kinase domain, C-terminal 4-EF hand motif containing calmodulin (CaM)-like domain (CLD), and a regulatory Junction domain, which connects these two domains. Most apicomplexan CDPKs follow a similar architecture, with some subtle differences [2,12]. The domain architecture and composition of Calcium Dependent Protein Kinase 7 (CDPK7) is very different from other CDPKs. It has two N-terminal EF-hand motifs and has a long linker, which connects them to a PH-domain adjacent to the kinase domain at the C-terminus [5,8] (Fig 1A). While it remains unclear if calcium regulates TgCDPK7 activity, TgCDPK7 interacts with phosphoinositides (PIPs) PI4P and PI(4,5)P2. Indeed, PIP interaction with the PH-domain is important for cellular localization of PfCDPK7 in *P*. *falciparum* [5]. There are only two major studies on CDPK7, which suggest that it is critical for the development of both *P*. *falciparum* [5] and *T*. *gondii* [8]. However, the underlying mechanisms are not understood, as its cellular targets and metabolic functions have remained unknown.

In the present study, we have dissected molecular processes that are regulated by TgCDPK7 in *T*. *gondii* intracellular tachyzoite development. Depletion of TgCDPK7 from *T*. *gondii* impaired trafficking of GPI anchored proteins. Quantitative phosphoproteomics revealed that TgCDPK7 may regulate phosphorylation of proteins putatively involved in lipid metabolism and protein/lipid trafficking. We demonstrate that it regulates phosphorylation of TgRab11a, which is critical for parasite division and its subcellular localization. Furthermore, TgCDPK7 was found to be involved in the regulation of phosphatidylethanolamine biosynthesis. A Glycerol-3-phosphate acyl transferase (GPAT) from *T*. *gondii*, which may contribute to PL biogenesis, was phosphorylated by TgCDPK7 and interacted with the kinase.

## Results

### TgCDPK7 regulates parasites division and protein trafficking

As mentioned above, TgCDPK7 (TGGT1_228750) does not have the canonical domain architecture of classical CDPKs. It has a PH domain adjacent to its kinase domain. Interestingly, a putative myrisotylation signal may be present at its N-terminus (Fig 1A).

A *T*. *gondii* line in which TgCDPK7 was conditionally knocked down was generated by using a TATi- based Tet-regulated system (S1A and S1B Fig). Our efforts to tag the N-terminus were unsuccessful suggesting that N-terminus may be critical for the function of this kinase. Phosphoproteomic analyses and most other studies were performed using this line in which TgCDPK7 was not tagged. The addition of anhydrotetracycline (ATc) resulted in a significant depletion of *TgCDPK7* mRNA within ~72h of treatment, as adjudged by Real Time PCR (S1C Fig). It was only recently that we obtained a C-terminally HA-tagged TATi-based iKD as also previously reported [8], which was used for some other studies (see below). As described below in detail, the addition of ATc to TgCDPK7-iKD parasites resulted in abrogation of the parasite lytic cycle, as revealed by plaque assays (S2 Fig). Assessment of intracellular growth rate assays revealed that TgCDPK7 depletion arrested parasite division (Fig 1B), which is consistent with previous studies [8] and ATc did not alter wild type ΔKu80 parasites (S1D Fig). Similar defects in parasite replication were observed in another independent clone of TgCDPK7-iKD parasites (S1E Fig).

Several defects caused due to TgCDPK7 depletion that were reported previously [8] were also observed, which included impaired centrosome duplication (S5F Fig), IMC and Golgi biogenesis (S5D and S5E Fig). Defects in microneme and rhoptry formation were also observed (S5B and S5C Fig), which was also consistent with previous studies [8]. However, there were no differences in the apicoplast biogenesis (S5A Fig).

In order to further assess the parasite morphology, immunofluoroscence assays (IFA) were conducted to monitor the localization of the GPI-anchored surface antigen SAG1 as well as ROM4, a transmembrane rhomboid protein which is also found at the parasite periphery/surface but is not GPI-anchored. SAG1, which is typically found uniformly distributed at the parasite plasma membrane in the absence of ATc, exhibited an unusual fragmented localization and was also found as punctate spots in TgCDPK7-iKD parasites and in some cases in the residual body in the presence of ATc (Fig 1C). This novel phenotype, which has not been reported, mainly became apparent by IFA in vacuoles that contained more than 4 parasites (Fig 1C and 1D). Interestingly, the localization of ROM4 (Fig 1C and 1D) as well as the glideosome protein GAP45 (Fig 3D) remained almost unchanged both small and larger vacuoles. Similar defects were also observed for another GPI-anchored protein SAG3 (Fig 1D). There was almost no change in SAG1 expression (S11 Fig). These studies suggested either trafficking of SAG1/3 to the parasite pellicle its GPI-anchoring was regulated by TgCDPK7.

### Identification of possible TgCDPK7-regulated parasitic processes by comparative phosphoproteomics

**Fig 2:**
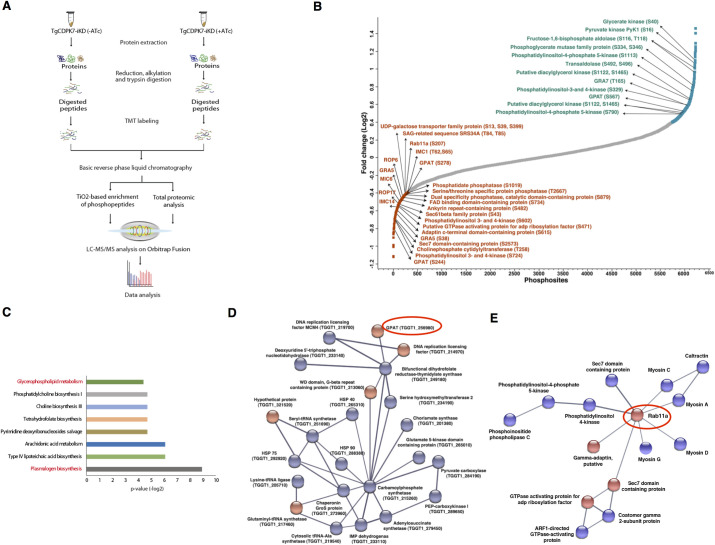
Comparative phosphoproteomics of TgCDPK7-iKD parasites. **A.** Schematic representation of the overall workflow employed for comparative proteomic and phosphoproteomic analysis. A TMT-labeling approach was used for TgCDPK7-iKD parasites (for details see Materials and Methods S1 Data 1.1). **B.** The phosphorylation fold-change ratios of phosphopeptides identified from TgCDPK7-iKD tachyzoites cultured in the presence or absence of ATc was normalized with respect to total protein fold-change. The ratios for all phosphopeptides from various replicates are provided in S1 Data, 1.1. The S-curve for normalized data is provided and some of the significantly altered phosphorylation sites belonging to key proteins (S1 Data, 1.2) are indicated. Representation of phosphosites and their corresponding abundance fold change in TgCDPK7 depleted parasites. Some of the differentially phosphorylated sites are highlighted in red and blue color, respectively and key target proteins that are relevant to the reported studies are indicated in green. **C.** Pathway analysis of proteins that exhibited reduced phosphorylation upon TgCDPK7 depletion and the possible metabolic pathways these proteins may regulate (S1 Data, 1.4). **D and E.** Protein-protein interactions were predicted between differentially phosphorylated proteins using STRING resource. The analysis exhibited high confidence protein-protein interactions between the candidate genes (S10 Fig and S1 Data, 1.6). Two major protein-protein interaction clusters involving TgRab11A (**D**) and GPAT protein (**E**) are illustrated.

In order to identify the downstream phosphorylation events and effectors of TgCDPK7, quantitative phosphoproteomic and proteomic analyses were carried out on TgCDPK7 mutant parasites in the presence and absence of ATc. For this purpose, TgCDPK7-iKD parasites were treated with ATc for 72h and subsequently tachyzoites were mechanically released from host cells. Tachyzoite lysates were prepared and used for proteomic studies. A Tandem Mass Tag (TMT)-labeling based approach (Fig 2A) that allows multiplexing capabilities for relative quantitative proteomic and phosphoproteomic analysis was used to measure differences in the phosphorylation state of proteins upon TgCDPK7 knockdown. The analysis was carried out in five biological replicates and resulted in identification of 13,090 unique phosphorylation sites (phosphosites) on 3,598 *T*. *gondii* proteins. Of these, 6228 phosphosites were consistently identified across multiple biological replicates and therefore, used for further analysis (Fig 2B and S1 Data). The phosphorylation levels of proteins were normalized with respect to protein abundance obtained from the proteomic analysis. The intensity of phosphopeptides in control and experimental samples were compared to identify the significantly altered phosphorylation sites. A p-value < 0.05 was used to filter phosphosites that exhibited consistent phosphorylation changes across replicates and a fold-change of 1.33 was used as a cut-off to define differentially phosphorylated sites, which was in accordance with our previous work [6] and were considered as significantly altered upon TgCDPK7 depletion. Fig 2B highlights some of the differentially phosphorylated proteins identified in the study (S1 Data, 1.2 and 1.3). In all, 301 phosphosites on 215 proteins exhibited reduced level of phosphorylation upon TgCDPK7 depletion. These data provided information regarding targets of TgCDPK7. Since TgCDPK7 was depleted for 72h, phosphorylation of some proteins may be affected nonspecifically as well, which is why independent studies were carried out for validation of some of the targets and pathways of interest (see below).

Interestingly, there were 463 phosphosites on 338 proteins that were identified to be significantly hyperphosphorylated upon TgCDPK7 depletion (S1 Data, 1.3). It is likely to be an indirect affect caused possibly due to TgCDPK7 activating other kinases, or suppressing protein phosphatases.

To focus on the putative targets of TgCDPK7 and their functions, we performed gene ontology and pathway analysis on proteins that exhibited hypophosphorylation in our study. The analysis was performed using the annotations available on ToxoDB, which suggested multiple pathways and biological processes enriched among the hypophosphorylated proteins (S1 Data, 1.4 and 1.5). Interestingly, proteins that are known to be involved in lipid biogenesis pathways like plasmalogen biosynthesis and glycerophospholipid metabolism were found to be hypophosphorylated upon TgCDPK7 depletion. Some of these enzymes are: A Glycerol-3-Phosphate-Acyl Transferase (GPAT), which initiates the synthesis of phosphatidic acid (PA) synthesis [i.e. central precursor for all phospholipid synthesis and important signal transducer was hypophosphorylated at two sites (S244 and S278) and hyperphosphorylated on one site (S567) (Figs 2B and 6A and S1 Data). Phosphatidate phosphatase (PAP), which catalyses the dephosphorylation of PA to form diacylglycerol (DAG, another central lipid precursor and signal transducer) was also found to be significantly hypophosphorylated (S1019) in the mutant. In addition, phosphoinositide (PIP) kinases like PI4K, which produce PIPs that regulate trafficking, were also affected. It is interesting to note that TgCDPK7 has previously been demonstrated to interact with PIPs [8]. Importantly, several proteins implicated in protein and/or lipid trafficking underwent significant changes in phosphorylation (Fig 2B and S1 Data, 1.2), which includes TgRab11a, Sec6 and Sec7 family proteins, a ARF-like GTPase [13,14]. To gain further insights into the process that may depend on TgCDPK7, protein-protein interaction (PPI) networks among differentially phosphorylated proteins (Figs 2D, 2E and S10) were predicted using the STRING database, which uses information from reported experiments and co-expression profiles and gene arrangement [15]. TgCDPK7 was predicted to be involved directly or indirectly with several of the identified proteins (S10 Fig). Two major modules that emerged from these studies were especially of interest as they related to defects in the observed phenotype in trafficking or PL biogenesis and comprised of proteins implicated in trafficking (Fig 2D) and enzymes involved in metabolic pathways (Fig 2E). These direct or indirect interactions among the differentially phosphorylated proteins provided important indicators to cellular processes that TgCDPK7 may modulate. Taking clue from these results, subsequent studies were performed to investigate the role of TgCDPK7 in aforementioned processes.

### A potential CDPK7 phosphosite on TgRab11a is critical for parasite division

**Fig 3:**
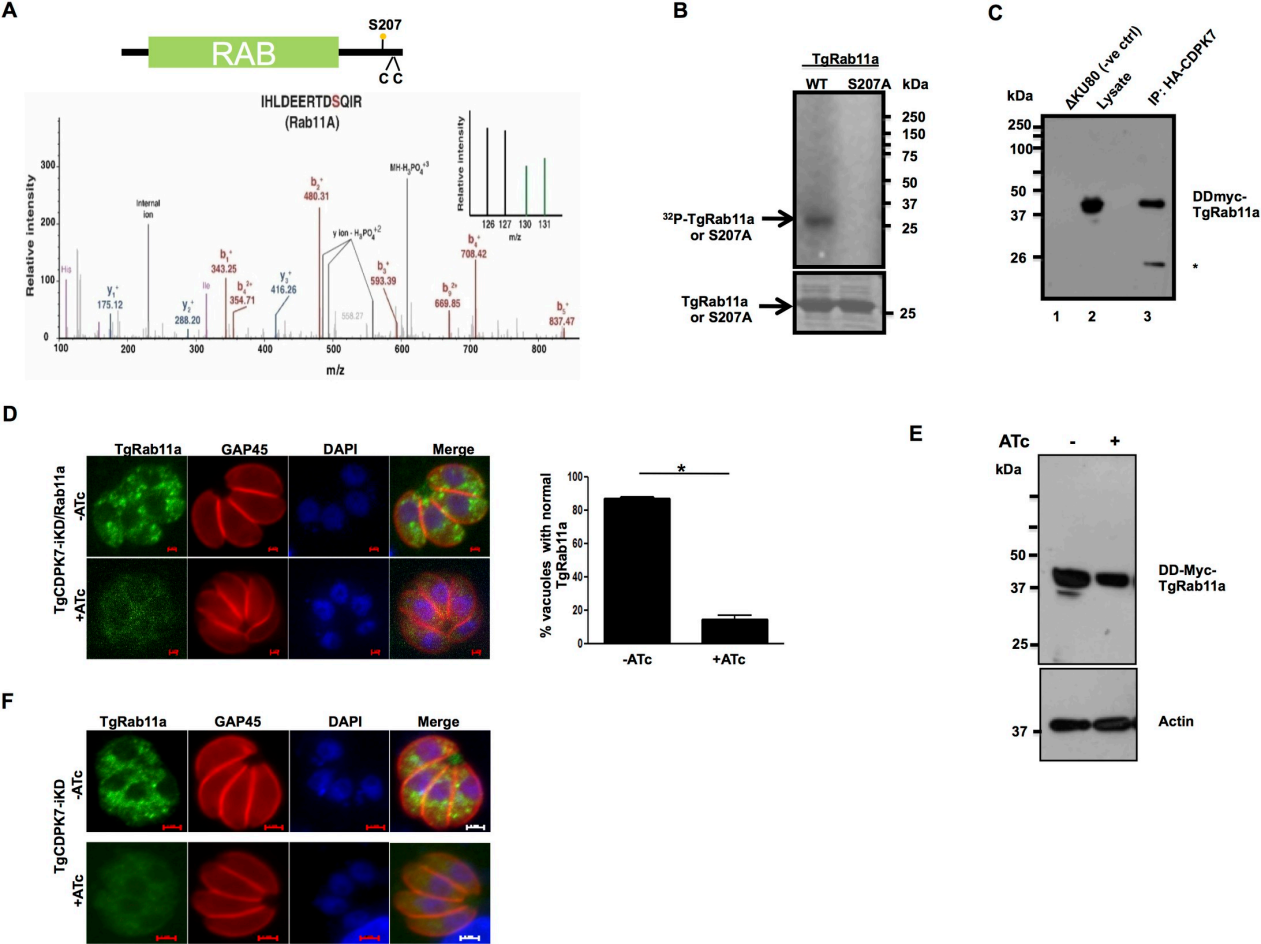
TgCDPK7 phosphorylates TgRab11a and regulates its cellular localization. **A.** Schematic illustrating TgRab11a domain architecture. The catalytic domain is in green, and the two cysteines (CC) that may be critical for lipid modification like prenylation are indicated. The phosphorylation of S207 was reduced upon TgCDPK7 depletion. MS/MS spectra is provided for the corresponding phosphopeptide. TMT channels highlighted in green in the inset represents independent biological replicates of TgCDPK7 depleted parasites and wild type samples are highlighted in black. **B.** A deletion mutant of TgCDPK7 (ΔTgCDPK7) that possesses the PH and kinase domain was expressed as a GST tagged protein and was used to phosphorylate recombinant 6xHis-TgRab11a or its S207A mutant *in vitro* using γ^32^P-labelled ATP. The reaction mixture was separated by SDS-PAGE and gel was used for phosphorimaging. **C.** DD-Myc-TgRab11a was ectopically expressed in the presence of Shield-1 in *T*. *gondii* parasites in which TgCDPK7 was tagged with HA (TgCDPK7-HA) at the C-terminus. TgCDPK7 was immunoprecipitated using anti-HA antibody. Western blotting was performed on TgCDPK7-IP (lane 3) from these parasites or ΔKu80 (negative control, lane 1) or whole cell lysate (lane 2) using anti-myc antibody. A band corresponding to DD-myc-TgRab11a was observed in TgCDPK7-IP. *, a possible non specific or break down product. **D.** DD-Myc-TgRab11a was ectopically expressed in *T*. *gondii* in TgCDPK7-iKD parasites. Shld-1 was added for 4h to stabilize the expression of TgRab11a and in addition ATc was added for 72h to deplete TgCDPK7. IFA was performed using anti-myc and anti-GAP45 antibodies, which revealed that punctate localization of TgRab11a was lost upon TgCDPK7 depletion. *Right Panel*, Quantification of vacuoles containing normal punctate TgRab11a staining, from experiments described in the left panel. Data are mean ± SE of three independent experiments and at least 200 vacuoles were counted for each condition (*, n = 3, p<0.001, t-test). **E.** DD-Myc-TgRab11a was ectopically expressed in *T*. *gondii* in TgCDPK7-iKD-HA parasites, ATc treatment was given for 72h and parasites were incubated with Shld-1 for 4h. Western blotting was performed using anti-myc antibody or anti-actin, which was used as loading control. **F.** IFA was performed using anti-TgRab11a antibody on untreated or ATC-treated TgCDPK7-iKD parasites. ATc treatment resulted in loss of punctate TgRab11a localization and was diffuse.

The fact that TgCDPK7 depletion impaired SAG1/SAG3 localization (Fig 1C and 1D) fits in well with observed defects in trafficking-related proteins. Phosphoproteomic data revealed that several proteins that are implicated in vesicular trafficking, which included TgRab11a, Sec7, Arf, PIP kinases, were differentially phosphorylated upon TgCDPK7 knockdown (Fig 2B). One of the PPI-networks that was predicted to be influenced by TgCDPK7 suggested TgRab11a to be at the hub of this network involving other proteins implicated in vesicular trafficking (Fig 2E). Therefore, it was important to investigate the regulation of TgRab11a by TgCDPK7. Apicomplexan parasites *T*. *gondii* and *P*. *falciparum* possess several members of small GTPases-Rabs, which have been implicated in critical parasitic functions [16–19]. It has been shown previously that the overexpression of a dominant negative form of TgRab11a causes defects in parasite division [17]. TgRab11a exhibited markedly reduced phosphorylation (~1.33 fold) at S207 (Figs 2B and 3A), which is at its C-terminal end [20]. The phosphorylation of recombinant (S3B Fig) TgRab11a by TgCDPK7 was observed in an *in vitro* kinase assay. In addition, when S207 was mutated to alanine, its phosphorylation was attenuated (Fig 3B). These data suggested that TgCDPK7 may directly phosphorylate TgRab11a in the parasite. Furthermore, efforts were made to determine if TgRab11a interacts with TgCDPK7 in the parasite. For this purpose, TgRab11a was expressed with a FKBP DD-domain and a myc tag at its N-terminus, which does not interfere with its function [17], in TgCDPK7-iKD parasite line (Fig 3E). The DD-domain is unstable when fused to proteins promotes their degradation and addition of its ligand Shield-1 stabilizes its expression, therefore is a useful tool for regulating protein expression [21]. In addition, TgCDPK7 was also tagged with HA at its C-terminus to facilitate its immunoprecipitation. Western blotting revealed that TgRab11a was pulled down in TgCDPK7-HA-IP (Fig 3C), which was also observed in reverse co-immunoprecipitation (S14C Fig). These data indicated that these two proteins interact in the parasite. TgCDPK7 seems to reside in punctate vesicular structures, while IFA studies did not reveal complete co-localization with TgRab11a vesicles, some co-staining was observed (S14D Fig). It is possible that these vesicles are dynamic and transiently contact TgRab11a-vesicles facilitating interaction and phosphorylation by the kinase, which is indicated by co-immunoprecipitation and kinase assay results.

**Fig 4:**
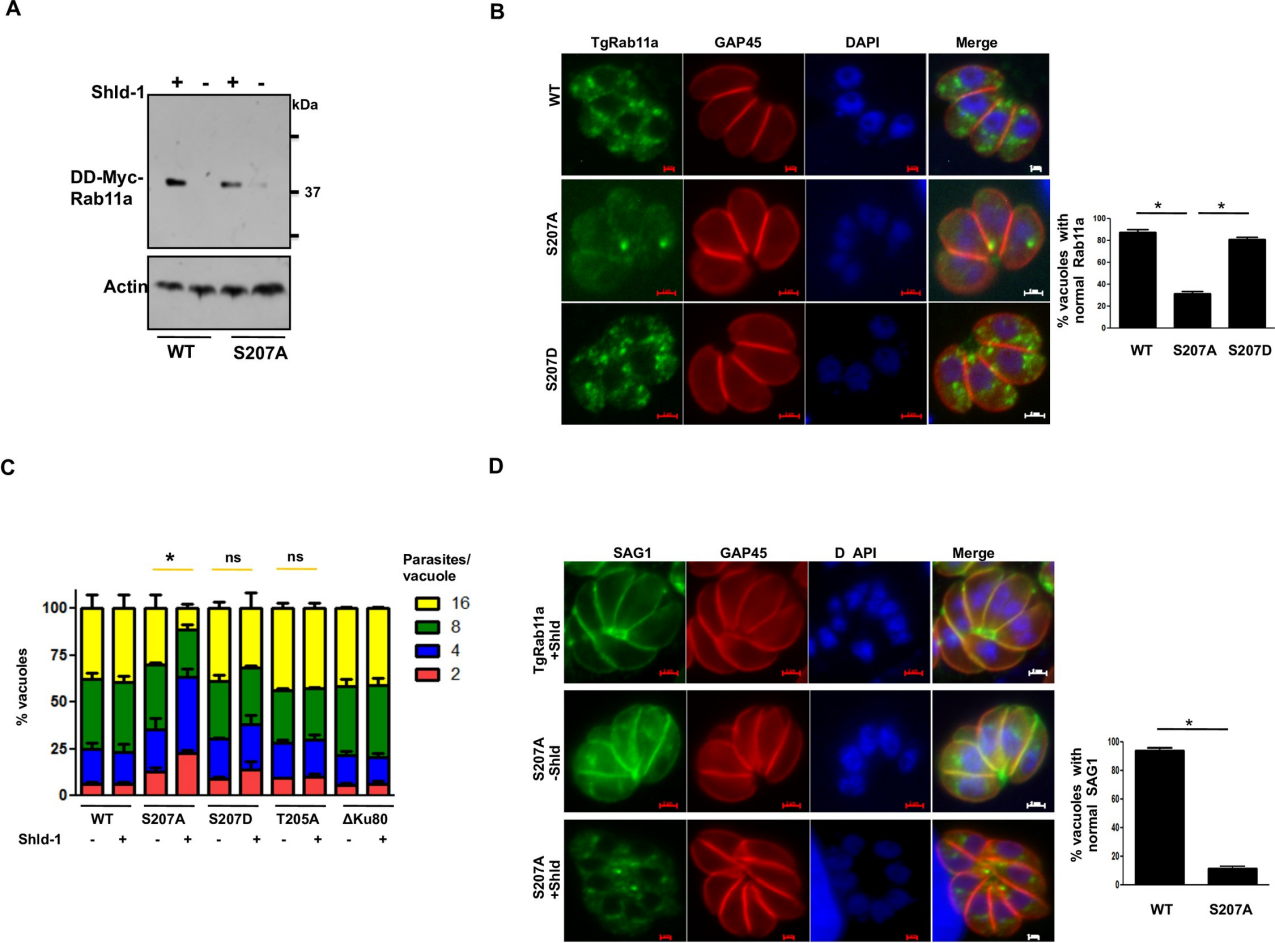
TgCDPK7-mediated phosphorylation of TgRab11a at S207 is critical for its function. **A.** DD-Myc-TgRab11a or its S207A mutant was ectopically expressed in *T*. *gondii* in ΔKu80 parasites. Shld-1 was added for 4h or parasites were left untreated. Western blotting was performed using anti-myc antibody and Actin was used as a loading control. **B.** Shld-1 was added for 4h to parasites ectopically expressing DD-Myc-TgRab11a or its S207A and S207D mutants in ΔKu80 parasites. IFA was performed using anti-myc and anti-GAP45 antibody. While WT TgRab11a and S207D exhibited punctate localization, S207A mutant was diffused in parasite cytoplasm in most cases. *Right Panel*, Quantification of vacuoles containing normal punctate TgRab11a or S207A/D staining, from experiments described in the left panel [Mean ±SE, *, n = 3 (WT and S207A) and n = 2 (S207D), p<0.0001, t-test]. **C.** DD-Myc-TgRab11a or its S207A, T205A, S207D mutants were ectopically expressed in *T*. *gondii*. Shld-1 was added for 16h and the number of parasites per vacuole was determined after additional 8h. Data represent Mean ± SE, n = 3 and at least 200 vacuoles were counted for each condition (ANOVA; WT, S207A, S207D: n = 3, T205A: n = 2. *, p<0.05, ns-not significant). **D**. IFA was performed on parasites expressing WT TgRab11a or its S207A mutant in the absence or presence of Shld-1 as described in panel B using antibodies against SAG1 and GAP45. *Right Panel*, Quantification of vacuoles containing normal SAG1 staining, which was at the parasite periphery, from experiments described in left panel. Data are mean ± SE of three independent experiments and at least 200 vacuoles were counted for each condition (*, n = 4, p<0.001, ANOVA).

Previous studies suggested that TgRab11a typically resides in punctate endosome-like compartments [17], which possibly allows it to play a role in trafficking of vesicles [16,17]. Therefore, the effect of TgCDPK7 depletion on TgRab11a localization was assessed. IFA revealed that in the absence of ATc TgRab11a exhibited punctate localization as previously described and mentioned above. In sharp contrast, TgCDPK7 depletion resulted in an almost complete loss of punctate staining and TgRab11a was diffuse in the parasite cytoplasm and its staining was weaker (Fig 3D). The expression of TgRab11a remained almost unaltered as assessed by immunoblotting (Fig 3E). Similar loss of punctate localization of endogenous TgRab11a was observed upon performing IFA with an anti-TgRab11a antibody [22] after depletion of TgCDPK7 (Fig 3F). Next, the role of S207 phosphorylation, which was significantly reduced upon TgCDPK7 depletion (Fig 3A and 3B), was investigated. For this purpose, WT or S207A TgRab11a was ectopically expressed in parental ΔKu80 parasites and Shld-1 was used to stabilize their expression (Fig 4A). IFA revealed punctate localization of TgRab11a as mentioned above. In contrast, S207A mutation resulted in almost complete abrogation of punctii and S207A mutant was diffused in the cytoplasm of ~60% parasites with occasional scattered dot-like aggregates (Fig 4B). In contrast, the localization of phosphomimetic S207D mutant was largely unchanged. The defect in the localization of S207A was more severe than the loss of phosphorylation of this site upon TgCDPK7 knockout as indicated by phosphoproteomics (Fig 3A). Limitations in precise estimation of phosphorylation fold changes in phosphoproteomic investigations may result in slight underestimation of fold decrease in phosphorylation. Therefore, phenotypic differences caused by S207A, which is totally devoid of phosphate at this site, are much higher. It is also possible, that in the absence of TgCDPK7 another kinase may compensate albeit not with similar efficiency.

We also monitored the localization of TgRab11b (S9B Fig), which exhibited phosphorylation but it remained unaltered upon TgCDPK7 depletion (S1 Data, 1.1). TgCDPK7 depletion did not cause any significant change in its localization (S9B Fig).

Collectively, these data indicated that TgCDPK7 regulates the localization of TgRab11a by mediating its phosphorylation at S207.

In order to ascertain the role of TgRab11a phosphorylation in parasite division, growth rate assays were performed by using parasites expressing either wild type TgRab11a or its S207A mutant. The ectopic expression of wild type (WT) TgRab11a did not cause any discernable difference in parasite replication upon Shld-1 addition. However, the addition of Shld-1 to S207A expressing parasites caused a significant attenuation of parasite division as more than 60% vacuoles contained 2–4 parasites (Fig 4C). There was no significant alteration in parasite replication when a phosphomimetic mutant S207D was overexpressed. These data suggested that parasites were arrested after 1–2 rounds of division and the defects caused by S207A mutant were dominant, as it caused these defects in the presence of endogenous TgRab11a. Overexpression of an alanine mutant (T205A) of neighboring T205 did not alter the parasite growth indicating that these defects were specific to S207 phosphorylation (Fig 4C). These data strongly indicated that the phosphorylation of S207 is critical for parasite replication.

Previous studies using a dominant negative mutant (N126I) of TgRab11a-which is catalytically incompetent as it keeps TgRab11a in GDP locked state-suggested that TgRab11a regulates parasite replication [16,22]. In addition, this mutant was recently shown to impair invasion and overexpression of this mutant impairs trafficking and secretion of dense granule (DG) proteins [22]. The overexpression of S207A mutant did not alter localization of DG protein GRA3 (S6D Fig) as well as microneme protein MIC2 (S6E Fig), which corroborated well with the fact that S207A mutant does not alter invasion (S7 Fig). S207A mutant caused aberrant Golgi and IMC biogenesis and centrosome duplication (S6A–S6C Fig), which was in sync with similar defects found upon TgCDPK7 depletion (S5D–S5F Fig).

Given that TgCDPK7 depletion caused defects in SAG1 localization to parasite pellicle (Fig 1B), the role of TgRab11a phosphorylation at S207 in this process was assessed. While WT TgRab11a overexpression did not alter SAG1 localization to the pellicle, SAG1 was not localized at the pellicle of S207A mutant overexpressing parasites. Instead, it was found mainly in the parasite cytoplasm (Fig 4D). The phosphomimic mutant S207D (S8A Fig) behaved like the wild type Rab11a as it did not cause any difference in SAG1 localization, which was also the case with T205A mutant (S8A Fig). Expectedly, S207A mutation did not alter TgCDPK7 localization, which is in punctii present in the cytoplasm (S9C Fig). These data indicated that the phosphorylation of TgRab11a at S207 is critical for trafficking of proteins like SAG1.

### TgCDPK7 is pivotal for the synthesis of phosphatidylethanolamine (PE) and PE supplementation reverts the defect in parasite replication

**Fig 5:**
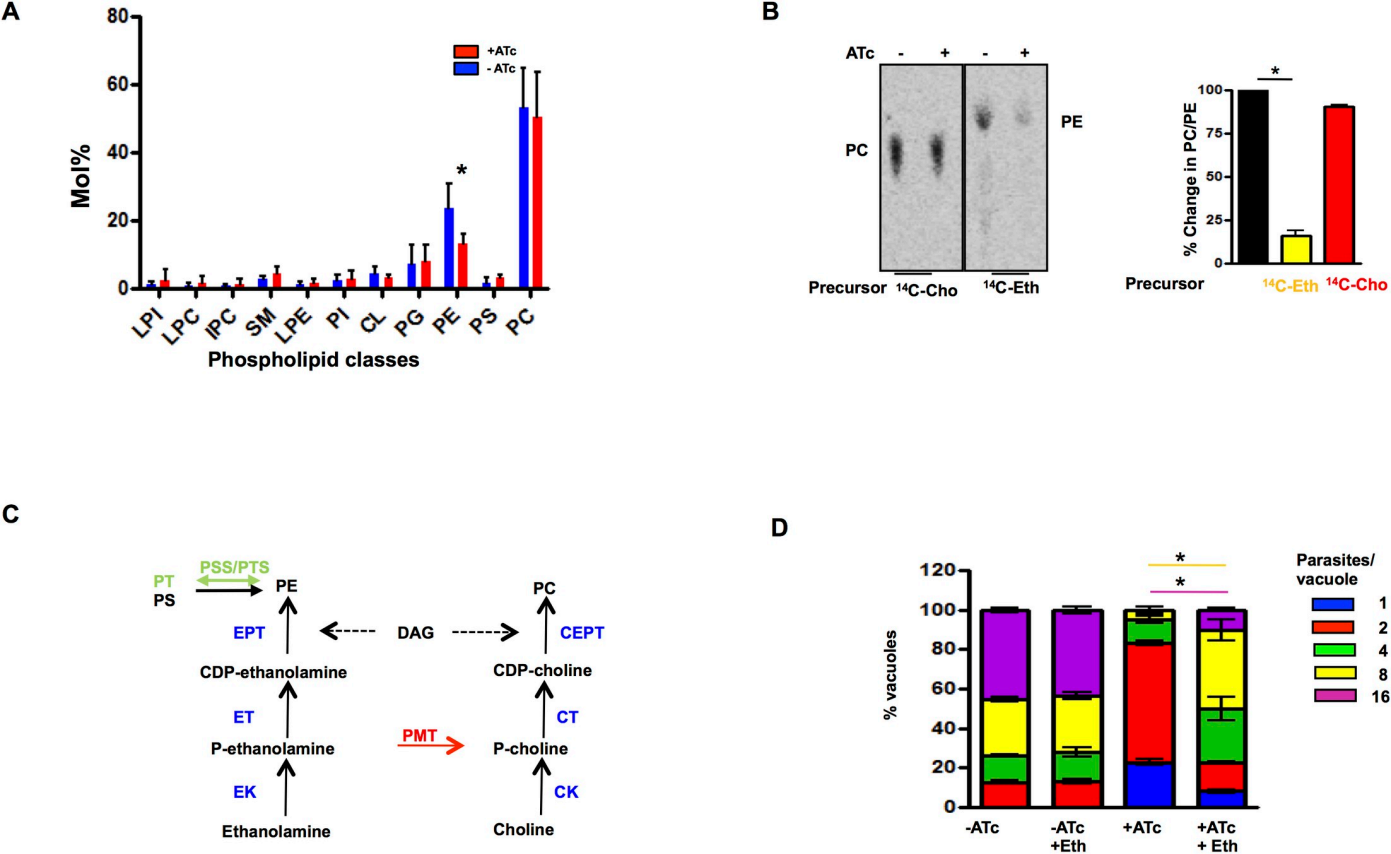
TgCDPK7 is involved in phospholipid metabolism. **A.** Total lipid isolated from TgCDPK7-iKD tachyzoites grown in the presence or absence of ATc and separated by HPTLC. After quantification by GC-MS and normalization with respect to cell number content relative to total fatty acids was determined. Phospholipid profiling showed the abundance of major phospholipid classes and points at the significant reduction of PE upon TgCDPK7 depletion (mean ± SE, *, n = 4, p<0.05, t-test). **B.** Metabolic labeling to monitor the synthesis of PC and PE. Extracellular TgCDPK7-iKD tachyzoites were incubated with ^14^C-Eth or ^14^C-Cho in the presence or absence of ATc. Subsequently, lipids were extracted and radiolabeled lipids were detected by phosphoimaging of TLC (Left panel). *Right Panel*, Radiolabeled spots corresponding to PE and PC (left panel) were quantitated by densitometry (right panel) and % change in PC and PE formation in ATc-treated parasites with respect to untreated parasites was calculated (mean ± SE, *n = 3, p<0.001, ANOVA). **C.** Metabolic pathways depicting the synthesis of PC and PE in *P*. *falciparum* and *T*. *gondii*. The enzymes conserved in two parasites are indicated in black. PMT is solely encoded by *P*. *falciparum*, is indicated in red and PTS/PSS involved in conversion of PE to PT or PS in *T*. *gondii* is shown in green [29]. **D.** TgCDPK7-iKD parasites were pre-incubated for 48h with ATc and were subsequently allowed to invade fresh HFFs in the presence or absence of ATc. In one case, 200 μM Eth was added to cultures prior to the addition of ATc. The number of parasites per vacuole was determined after 24h. Data represent mean ± SE, n = 3 and at least 200 vacuoles were counted for each condition (ANOVA, *, p<0.001 for 8/16 parasites/vacuole).

One of the highlights of phosphoproteomics data was a significant change in the phosphorylation of proteins/enzymes involved in phospholipid (PL) and fatty acid FA metabolism upon TgCDPK7 depletion (Fig 2B and 2C). In addition, a PPI-module which involves these proteins was predicted through *in silico* analysis (Fig 2D). Given these observations, it was worth investigating if there was a bearing on PL biogenesis in *T*. *gondii* tachyzoites. To this end, mass spectrometry-based lipidomic analyses was conducted on lipids extracted from TgCDPK7-iKD parasites grown either in the absence or the presence of ATc. The analysis of parasite phospholipid composition revealed that there was a significant reduction in total phosphatidylethanolamine (PE) content, whereas no other phospholipid class was affected upon TgCDPK7 depletion (Fig 5A). It is important to note that PE is the second major PL in *T*. *gondii* [23] and the parasite has multiple pathways to synthesize PE: the Kennedy Pathway involves use of ethanolamine as precursor and GPAT facilitates the generation of phosphatidic acid (PA), which is converted to DAG and is further used for PE formation; Phosphatidylserine (PS) is also used via the PSD pathway for PE generation [24] that occurs in mitochondria (Fig 5C). To confirm lipidomic data, the *de novo* synthesis of PC and PE via the parasite Kennedy pathway (Fig 5C) was monitored by metabolic labeling. For this purpose, ^14^C-Ethanolamine and ^14^C-Choline were used as precursors of PE and PC in intracellular parasites, respectively. Total lipids were extracted and labeled lipids were detected and quantified by High Precision-Thin Layer Chromatography (HPTLC) (Fig 5B). We could not detect any significant differences in PC formation upon TgCDPK7 depletion (Fig 5B). In contrast, PE synthesis via the Kennedy pathway was drastically reduced (~70%) upon TgCDPK7 depletion (Fig 5B), which correlated well with lipidomic results (Fig 5A).

Previous studies have indicated that exogenously added Ethanolamine (Eth) can be utilized by the Kennedy pathway, i.e. CDP-Eth pathway, for PE synthesis in *T*. *gondii* [24]. Since the disruption of TgCDPK7 strongly impairs the synthesis of PE, the effect of supplementing parasites with PE precursor Eth was tested. To this end, TgCDPK7-iKD parasites were cultured either in the normal culture medium, or medium that was supplemented with additional ethanolamine. To determine the effect of Eth supplementation in greater detail, we quantified the number of parasites/vacuoles in all four conditions: -ATc, -ATC+Eth, +ATc, +ATc+Eth. As previously demonstrated, the reduction of TgCDPK7 expression by the addition of ATc to the cultures of TgCDPK7-iKD lead to defects in intracellular development (+ATc). However, when supplemented by Eth (+ATc +Eth), there was a partial but significant restoration of the parasite growth, as reflected by an increase in the number of vacuoles that had significantly more number of parasites (Fig 5D). Studies performed on another independent clone of TgCDPK7-iKD parasites revealed similar defects in replication upon TgCDPK7 depletion, which was significantly reverted upon Eth supplementation (S1E Fig). Similar observations were made in plaque assays as Eth addition partially restored plaques, which were lost upon TgCDPK7 depletion, whereas excess choline had no effect on parasite growth (S2 Fig). Interestingly, ethanolamine supplementation only partially restored SAG1 mislocalization in TgCDPK7 depleted parasites (S15 Fig). Since ethanolamine head group can be used for GPI synthesis [25], it is possible that the defective PE synthesis might contribute to this aberrant SAG1 localization as SAG1 is a GPI-anchored protein. Collectively, these studies strongly indicated that TgCDPK7 regulates biogenesis of PE, which is critical for parasite replication.

We also determined the total FA as well as PC/PE composition of the parasite. Upon TgCDPK7 depletion (+ATc), there was a significant reduction in total FA C16:0 and C18:1 in the TgCDPK7 mutant whilst there was a concomitant increase of long polyunsaturated FA (PUFA), C20:1, C20:4 and C20:5 (S12A Fig). Fluctuations in -ATc parasites PE C16:0 and C18:1 containing FA was observed, which makes the mean statistically not significant (S12B Fig). However, trends similar to the ones in total FA (S12A Fig) were observed in individual replicates. A similar analysis of PC-FA content suggested only a modest change in C16:0, C18:1 FA composition of PC (S12C Fig), which correlated with almost no change in the level of this PL (Fig 5A). Due to fluctuations in PE-FA content in untreated parasites, it is difficult to make strong conclusions but these results do hint at the possibility of altered FA composition of PE in ATc treated parasites.

Given that PE biogenesis was altered upon TgCDPK7 depletion, we tested if TgRab11A somehow regulates PL biogenesis. There was no significant change in PC/PE biogenesis upon overexpression of S207A mutant (S13A Fig). Moreover, Eth supplementation did not cause a significant reversal in growth defect exhibited by S207A mutant (S13B Fig) Therefore, these data suggested that Rab11a-S207 phosphorylation may not contribute to PL biogenesis.

### TgCDPK7 phosphorylates and interacts with GPAT

**Fig 6:**
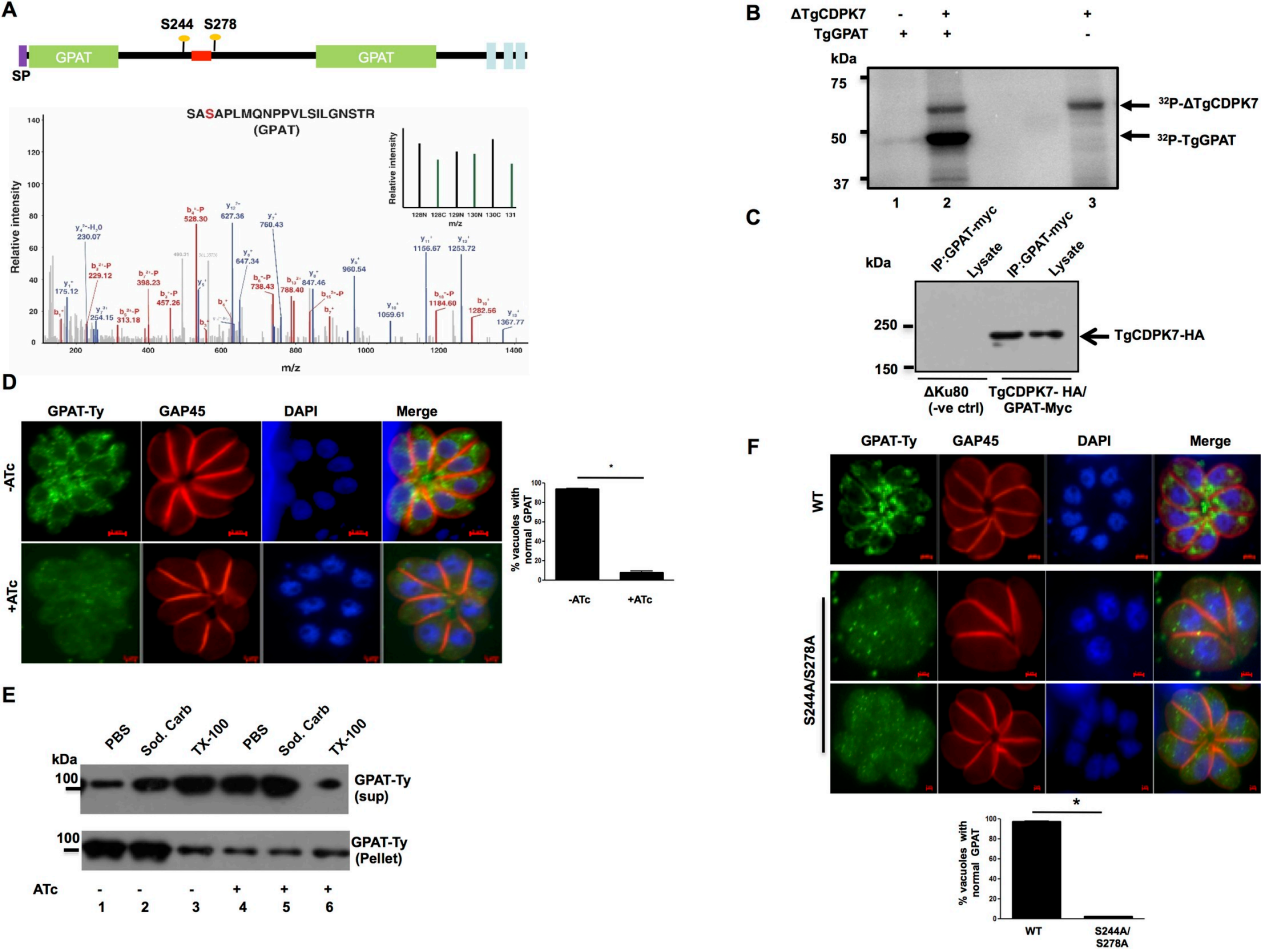
TgCDPK7 interacts with TgGPAT and regulates its localization. **A.** Domain architecture of TgGPAT (ToxoDB ID: TGGT1_256980) indicating N-terminal signal peptide and three transmembrane domains. The domain analysis suggested that its GPAT domain is split due to the presence of an insert. Multiple sites (S244, S278) on GPAT protein were identified to be hypophosphorylated upon TgCDPK7 depletion that were located in the insert. The location of the myc/Ty tag introduced in TgGPAT between aa 256 and 257 is indicated in red. *Bottom Panel*: MS/MS spectra for S278, one of the sites which was hypophosphorylated upon TgCDPK7 depletion (green bars). **B.** A deletion mutant of TgCDPK7 (ΔTgCDPK7) that has the PH and kinase domain was used to phosphorylate recombinant fragment of GPAT as a GST tagged protein *in vitro* using γ^32^P-labelled ATP. The reaction mixture was separated by SDS-PAGE and gel was used for phosphorimaging. Autophosphorylation of the kinase was also observed. **C.** A myc-tag was introduced in TgGPAT gene after a.a 256 using CRISPR-Cas9 in C-terminally HA tagged TgCDPK7 parasite line. Parasite lysates were prepared from these or parental (ΔKu80) parasites and were used for IP with anti-myc antibody. Subsequently, Western blotting was performed on total lysate and GPAT-myc IP using anti-HA antibody to detect TgCDPK7-HA. A band corresponding to TgCDPK7-HA was observed only in GPAT-myc IP from transgenic parasites, which was the same size as the band in the whole cell lysates. No band was observed in the case of ΔKu80 parasites (negative control). **D.** GPAT was Ty-tagged at endogenous locus as described in panel A in TgCDPK7-iKD parasites (TgCDPK7-iKD/GPAT-Ty). ATc was added for 72h to deplete TgCDPK7 followed by IFA for GPAT-Ty. There was a significant change in the localization of GPAT from perinuclear ER like compartment (-ATc) to predominantly cytoplasm upon TgCDPK7 depletion (+ATc). *Right Panel*, Quantification of vacuoles containing normal perinuclear GPAT localization, from experiments described in the left panel. Data are mean ± SE of three independent experiments and at least 200 vacuoles were counted for each condition (*, n = 3, p<0.001, t-test). **E.** TgCDPK7-iKD/GPAT-Ty parasites were treated with ATc as described in panel D. Subsequently, parasite pellets were isolated and used for extracting proteins in PBS, sodium carbonate pH 11.0 or 1% Triton X-100. The supernatant (sup) or pellet fraction was electrophoresed and subjected to Western blotting using anti-Ty antibody to detect GPAT. **F.** C-terminal Ty-tagged full length TgGPAT or its S244A/S278A mutant were transiently overexpressed in ΔKu80 parasites. After 48h, parasites were fixed and IFA was performed using anti-Ty and anti-GAP45 antibodies. *Bottom Panel*, Quantification of vacuoles containing normal ER-like perinuclear TgGPAT or S244A/S278A staining, from experiments described in the right panel. Data are mean ± SE of three independent experiments and at least 200 vacuoles were counted for each condition (*, n = 3, p<0.001, t-test).

**Fig 7:**
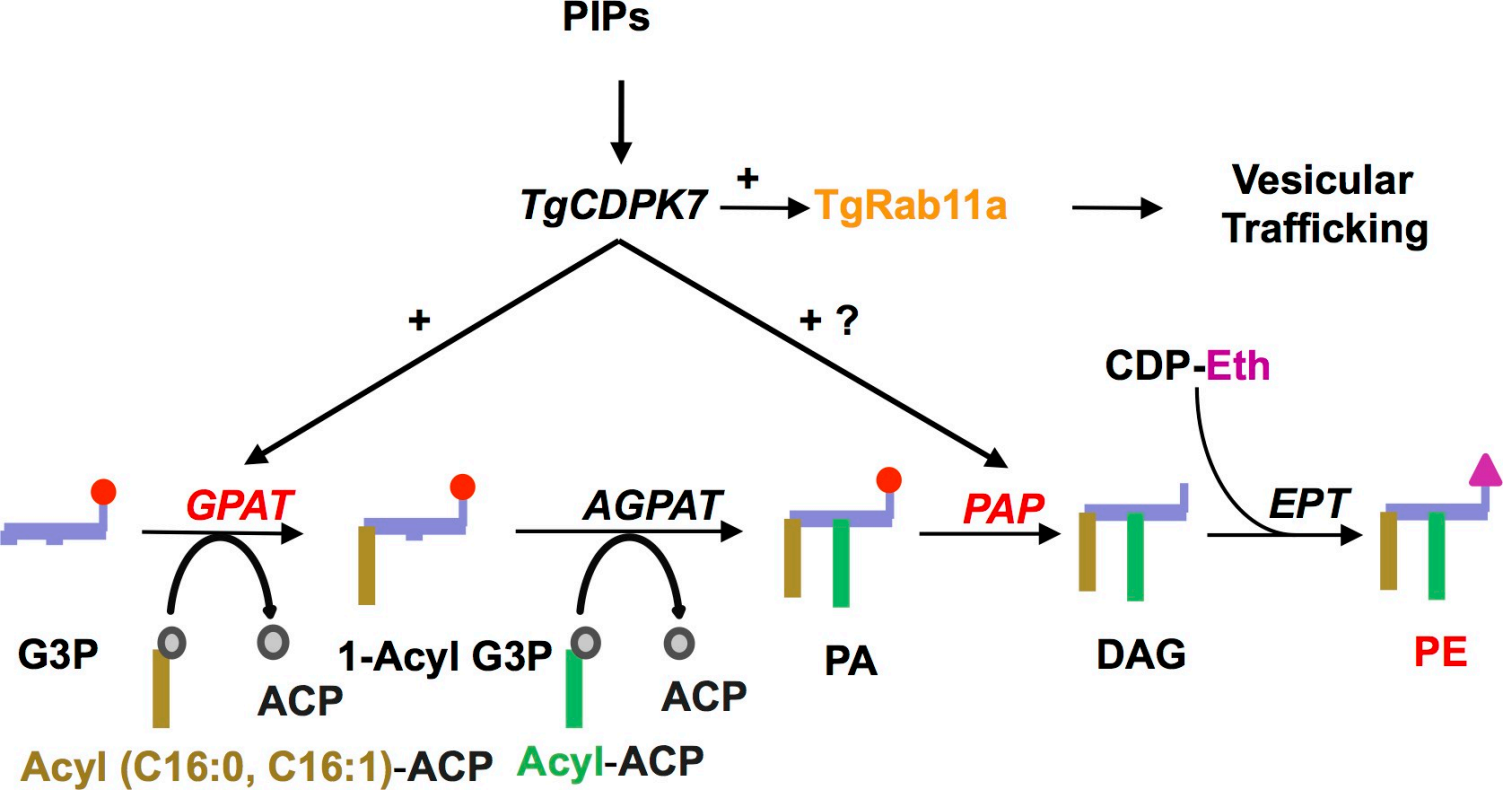
A model for TgCDPK7 signaling. TgCDPK7, which interacts with PIPs [8], targets trafficking of key proteins like GPI linked proteins like SAG1/3 via its ability to facilitate the phosphorylation of proteins like TgRab11a. It regulates PE metabolism in the parasite possibly via its ability to regulate enzymes involved in PL metabolism like GPAT and PAP and these events are critical for the division of *T*. *gondii*.

Several enzymes implicated in PL biogenesis exhibited reduced phosphorylation upon TgCDPK7 depletion (Fig 2B and 2D). It was interesting to note that TgGPAT exhibited altered phosphorylation at several sites upon TgCDPK7 depletion (Figs 2B and 6A). TgGPAT was of particular interest as it is critical for PA formation, which is further utilized for biogenesis of PLs like PC/PE (Fig 7). However, the function of this GPAT has not been reported in *T*. *gondii*. GPAT was phosphorylated by TgCDPK7 *in vitro* (Fig 6B). TgGPAT has a signal peptide at its N-terminus and has three predicted transmembrane domains at the C-terminus (Fig 6A), which has close similarity to ER GPAT from *P*. *falciparum* [26]. Interestingly, domain analysis suggested that its catalytic GPAT domain is split due to a long insertion, which seems to be specific to *T*.*gondii* and the modulated phosphorylation sites are also present in the insertion. In order to ascertain if these proteins interacted in the parasite, efforts were made to tag endogenous TgGPAT at C-terminus but were not successful. However, a myc or Ty-tag was successfully inserted in the GPAT domain using CRISPR-Cas9 at TgGPAT endogenous locus (Fig 6A) in TgCDPK7-iKD parasites. Using these parasites, we were able to demonstrate that TgCDPK7 and TgGPAT co-immunoprecipitated indicating their interaction in the parasite (Figs 6C and S14C). Next, we tested if TgGPAT localization was dependent on TgCDPK7 by performing IFAs. TgGPAT was localized to compartments that were perinuclear and possibly the ER (Figs 6D and S14A). TgCDPK7 depletion caused a significant change in the localization of TgGPAT, which was diffuse in parasite cytoplasm (Figs 6D and S14A) without causing change in its expression (S14B Fig). Subcellular fractionation revealed that most of the GPAT was soluble in Triton X-100 (Fig 6E, lane 3,top panel) whereas only small amounts were found soluble in PBS or sodium carbonate and most of it was found in the insoluble pellet (Fig 6E, lane 1 and 2, top panel). In contrast, when TgCDPK7 was depleted TgGPAT was mostly solubilized in PBS or sodium carbonate with very small amounts in the pellet fraction (Fig 6E, lane 4 and 5). These observations indicated that TgCDPK7 depletion prevented its localization to the membrane of the ER and correlated well with the IFA results. These studies suggested that TgCDPK7 regulates TgGPAT localization in the parasite. Our attempts to generate phosphorylation site (S244, S278) alanine mutant of TgGPAT using CRISPR-CAS9 failed three times. While it did not allow further investigation, this possibly suggested the importance of phosphorylation of this site in the function of this enzyme. The wild type GPAT or a mutant in which two phosphorylation sites S244 and S278 were mutated to alanine were transiently overexpressed in *T*. *gondii*. IFA revealed that GPAT localized to perinuclear ER-like compartment (Fig 6F) as described above (Fig 6D) for endogenously tagged GPAT. In contrast, the phosphomutant was diffuse in the parasite implicating a role of these phosphorylation sites in GPAT localization (Fig 6F). Collectively, these studies suggested that TgCDPK7 regulates TgGPAT localization in the parasite. Since TgGPAT is likely to be important for PL biogenesis at the ER, which is also suggested by our preliminary studies, it is reasonable to suggest that the mislocalization of TgGPAT is likely to impair its function, which possibly contributes to reduced PE levels in TgCDPK7 depleted parasites (Fig 5A and 5B).

## Discussion

CDPK7 is an atypical member of the Apicomplexan CDPK family and there is currently no evidence whether it is regulated by calcium. However, previous studies on PfCDPK7 and TgCDPK7 suggested that it interacts with PIPs via its PH domain [5,8]. Consistent with a previous study [8], we found that its depletion caused defects in parasite replication (Fig 1B). Comparative phosphoproteomic studies directed at identifying TgCDPK7 targets showed that effectors of protein trafficking like TgRab11a exhibited altered phosphorylation states (Fig 2B and 2E). These observations suggested regulatory networks involving TgCDPK7, PIP signaling and vesicular trafficking (Fig 7). A key member of Rab GTPase family-TgRab11a- exhibited differential phosphorylation upon TgCDPK7 depletion. TgCDPK7 mediated phosphorylation regulated its localization to vesicular compartments in the parasite. It was interesting to note that previously a GDP-locked mutant N126I of TgRab11a, which is defective in catalytic activity, caused defects both in invasion as well as intracellular replication [17]. Recently, this mutant was shown to impair the localization of GRA3 to dense granules and its secretion [22], which is relevant for its role in invasion. We did not find any defects caused by S207A in GRA3 localization (S6D Fig) as well as host invasion (S7 Fig). The fact that the mutation of TgRab11a S207 to A had a dominant impact on parasite division suggested that the phosphorylation of this site might be critical for this process. Since S207A mutant overexpression does not cause any apparent defects in invasion (S7 Fig), the phosphorylation of S207 may not be critical for invasion and is mainly involved in parasite replication. Several defects caused by TgCDPK7 in centrosome, Golgi and IMC biogenesis, which were reported previously [8] and also found in the present study (S5D–S5F Fig), were also observed upon S207A mutant overexpression (S6 Fig) suggesting that TgCDPK7 may regulate these events via phosphorylation of TgRab11a.

Rab11a has been implicated in recycling membrane material during cell division in other organisms [27]. Interestingly, S207A mutant overexpression also impaired SAG1 localization to the pellicle (Fig 4D). These data were consistent with previous studies that implicated TgRab11a in transport vesicles from endosome like compartments to the plasma membrane and regulate SAG1 localization to the parasite membrane during cytokinesis [17] and provide novel insights into the role of phosphorylation in the function of TgRab11a. Given that TgCDPK7 also regulates SAG1 trafficking to the membrane (Fig 1C), it is reasonable to conclude that it may achieve this via TgRab11a phosphorylation. Eth supplementation did not reverse the loss of pellicle localization of SAG1 in S207A overexpressing parasites (S8B Fig), which is consistent with the fact that PE biogenesis was almost unaltered in these parasite (S13A Fig). However, ethanolamine supplementation lead to partial restoration of SAG1 localization at the pellicle (S15 Fig) in TgCDPK7 depleted parasites in which PE biogenesis is impaired (Fig 5A and 5B). As mentioned above, PE is known to contribute to GPI biogenesis [25]. TgCDPK7 may regulate the localization of GPI-anchored proteins like SAG1/3 by independent mechanisms: it may regulate their trafficking via Rab11a; its role in PE biogenesis may facilitate synthesis and or trafficking of GPI via which proteins like SAG1/3 anchor to parasite membrane.

A human homologue of TgRab11a is also phosphorylated [28] but lacks the phosphorylation site complementary to S207 of TgRab11a, which is closer to the C-terminal lipid modification site (Fig 3A). S207 is also not conserved in the *P*. *falciparum* homologue PfRab11a. Subcellular fractionation did not reveal any significant changes in TgRab11a solubility upon TgCDPK7 depletion (S9A Fig). Apart from the conserved CC motif important for lipid modification, the C-terminal tail of Rabs especially Rab11 family is very divergent. Interestingly, the corresponding site was found only in *Neospora*, *Besnoitia*, *Cytoisaspora*, *Eimeria* and *Cryptosporodium*. Although further experimentation is needed, it is possible that TgCDPK7-mediated phosphorylation may be important for association with other factors, which may be important for its correct localization.

As discussed above, we found that TgRab11a PE biogenesis independent of Rab11A phosphorylation (S13 Fig), which indicated that it might be via independent mechanisms. One of the features of comparative phosphoproteomics was altered phosphorylation of key enzymes implicated in the biogenesis of PLs. Strikingly, TgCDPK7 depletion impaired PE formation, which was critical for parasite development and division (Fig 5A and 5B). PE can be synthesized in the ER of *Toxoplasma* via the CDP ethanolamine pathway, as well as in the mitochondria via decarboxylation of PS [29]. While lipidomic studies, which assessed total PE levels, indicated a significant (~35–40%) decrease upon TgCDPK7 depletion (Fig 5A), metabolic labeling studies which estimated PE formation by Kennedy or CDP-ethanolamine pathway indicated much higher decrease suggesting that ethanolamine was not properly utilized for PE biogenesis via this pathway (Fig 5B). The apicoplast contributes mainly to the synthesis of C12:0, C14:0 FA via its prokaryotic type II fatty acid synthesis pathway [30,31]. These FA chains can either (i) be transferred to G3P by the apicoplast plant-like glycerol 3-phosphate acyltransferase (TgATS1) [30,32] to generate LPA, which is exported towards the ER for the bulk PL synthesis or (ii) directly exported to the ER to be elongated to form longer FA chains including C16:0, C16:1, and C18:1 [24,33]. Consistent with this, the knock down of apicoplast localized glycerol-3-phosphate acyltransferase (TgATS1) caused a major reduction of PC and PI (~60–70%), whereas PE levels were also reduced to a lesser degree (~40% reduction) [30]. Very interestingly, our studies indicated that glycerol-3-phosphateacyltransferase (GPAT), a counterpart of ATS1 of eukaryotic origin had several phosphorylation sites effected by the depletion of TgCDPK7. TgGPAT was phosphorylated by TgCDPK7 directly in an *in vitro* kinase assay (Fig 6B). Moreover, GPAT exhibited interaction with TgCDPK7 in the parasite. Interestingly, TgCDPK7 depletion caused mislocalization of TgGPAT to parasite cytoplasm. These data suggested that phosphorylation and/or interaction via TgCDPK7 is critical for its localization to the ER. Given that TgCDPK7 is proximal to ER, one of the possibilities is that TgGPAT phosphorylation is co-translational which aids its retention and anchoring in the ER. This TgGPAT has not been characterized thus far but it has features of ER- resident TgGPAT, which possess a N-terminal signal peptide and transmembrane domain helices at C-terminal (Fig 6A). Therefore, its mislocalization upon TgCDPK7 knockdown is likely to have a bearing on FA/PL synthesis by at the ER, which was possibly reflected by a significant decrease in C16:0, C18:0 FA upon TgCDPK7 depletion. The cellular localization of GPAT was unaffected by TgRab11a S207A mutant (S6F Fig). Importantly, previous studies on *P*. *falciparum* homologue of GPAT (PfGPAT) revealed that it is a putatively essential ER-based protein that catalyses the synthesis of LPA, preferentially using C16:0 and C16:1 as substrates [26]. Here, we show that the overall FA content in the TgCDPK7 mutant may be depleted in C16:0 and C18:1, whilst long and likely scavenged PUFA (C20:3, C20:4, and C20:5) were increased (S12A Fig). It is possible that PE-FA content may also be altered in a similar manner but fluctuations in replicates prevent us from making a strong conclusion at this point. Phosphoproteomics also revealed that a PAP is also altered in its phosphorylation levels upon TgCDPK7 depletion. Therefore, a plausible explanation for this is that TgCDPK7 regulates the ER-based TgGPAT and possibly PAP to stimulate the utilization of free fatty acids that are directly exported from the apicoplast and elongated in the ER to be incorporated into PA, and this PA could then be utilized to make PE. Collectively, these data strongly hinted that TgCDPK7 might regulate PE biosynthesis by modulating key enzymes in the relevant pathways.

## Materials and methods

### Materials

All chemicals, oligonucleotides were purchased from Sigma-Aldrich unless indicated otherwise. Restriction enzymes were purchased from NEB. Cell culture reagents like Roswell Park Memorial Institute medium (RPMI) 1640, Dulbecco modified Eagle medium (DMEM), fetal bovine serum (FBS), trypsin, antibiotic-antimycotic and Albumax II were bought from Gibco (Thermo Fisher) USA. Antibodies were ordered from Santa Cruz Biotechnology (mouse anti-β-actin, sc-47778, 1:1,000 for Western blotting), mouse and rabbit anti-HA (Y-11, sc-805, 1:500 for WB and 1:200 for IFA) and Roche (anti-anti-GFP, 1:200 for IFA). anti-TgCentrin-1 was purchased from Kerafast. *Toxoplasma gondii* antibodies anti-GAP45 (rabbit, 1:10000), anti-SAG1 (mouse, 1:5000), anti-SAG3 (mouse, 1:200), anti-ROM4 (rabbit, 1:5000) were gifts from Prof. Dominique Soldati. anti-TgRab11a (mouse, 1:500) was kindly gifted by Dr. Sabrina Marion. For immunoblot analyses, horseradish peroxidase-labeled secondary goat anti-rabbit/mouse antibodies (Molecular Probes) were used. For immunofluorescence assays (IFA), the secondary antibodies Alexa Fluor 488- and Alexa Fluor 594-conjugated goat anti-mouse/rabbit antibodies (Molecular Probes) were used. PCR primers (S1 Table) were synthesized by Sigma Aldrich.

### Proteomics studies

#### Cell lysis and protein extraction

For the analysis of TgCDPK7-iKD parasites, ATc treatment was given for 72h to knock down TgCDPK7 after which tachyzoites were mechanically released from host cells. Host cell debris was removed by low speed centrifugation at 300g for 5 min and parasites were collected from supernatant. Subsequent to washing and separation of host cells, tachyzoite pellets of untreated or ATc treated TgCDPK7-iKD parasites was used. Pellets of equal number of mutant and wild type parasites were suspended in lysis buffer (8M Urea, 1mM sodium orthovandate, 1 mM sodium fluoride, 2.5 mM sodium pyrophosphate and 1 mM β-glycerophosphate). The parasite pellets were sonicated and proteins were extracted as described previously [6]. In brief, equal amounts of protein were processed for comparative phosphoproteomic and total proteomic analysis. Proteins were subjected to reduction, alkylation and trypsin digestion. Resulting peptides were dried and processed further for TMT labeling as per the manufacturer’s protocol (Thermo Fisher Scientific).

The analysis was carried out on five independent biological replicates. Each biological replicate was further analysed in technical duplicates on mass spectrometer to minimize the false positives.

#### TMT labeling

Peptides obtained from TgCDPK7 mutant and wild type *T*. *gondii* parasites were labeled with TMT mass tags as per the manufacturer’s instructions. Approximately, 3 mg peptides from mutant and wild-type parasites was labeled and the labeling details are provided in the S1 Data, 1.7. Labeling efficiency was estimated by analyzing 2 μg equivalent of labeled peptides on mass spectrometer. Labeled peptides were then pooled, dried and processed for fractionation.

#### Fractionation of the peptides and enrichment of phosphopeptides

Pooled peptides were fractionated using basic Reverse Phase Liquid Chromatography as described earlier [6]. The fractionation was carried out on Xbridge C18 5 μm 250 × 4.6 mm column (Waters Corporation, Milford, USA) using Agilent 1100 binary pump (Agilent Technologies, Santa Clara, USA). A gradient of 5 to 60% solvent B (10 mM triethyl ammonium bicarbonate, pH 8.5 in 95% acetonitrile) for over 60 min was used with 1ml flow rate per minute. The fractionated peptides were eventually concatenated into 12 fractions and vacuum dried. An aliquot from each fraction was taken out for total comparative proteomic analysis and remaining sample was further processed for TiO2-based enrichment of phosphopeptides. Peptides and titansphere beads (GL Sciences, Japan) were mixed in 1:1 ratio and incubated on rotor for 1 h. Unbound peptides were washed away using 80% acetonitrile in 3% TFA. Phosphopeptides bound to TiO2 beads were eluted using 4% ammonia solution. Each fraction was cleaned using C18 stage tips followed by LC-MS/MS analysis.

#### Data analysis

LC-MS/MS analysis of each fraction was carried out on Orbitrap Fusion mass spectrometer interfaced with Proxeon Easy-nLC 1000 system (Thermo Scientific, Bremen, Germany) in replicate runs. Each fraction was reconstituted in 20 μl of 0.1% formic acid and peptides were loaded on 2 cm long pre-column packed with C18 material. Peptides were then resolved on 25 cm long analytical column (75 μm in diameter, C18, 3 μm particle size) using a linear gradient of solvent B (7% to 30%) over 110 minutes. MS scans (400–1600 m/z) were acquired on Orbitrap mass analyzer with a mass resolution of 120,000 at 200 m/z. AGC target value was set to 200,000 with ion injection time of 50 ms. Precursor ions were fragmented using higher-energy collisional dissociation (HCD) with 34% normalized collision energy. MS/MS scans were acquired on Orbitrap mass analyzer with 30,000 mass resolution at 200 m/z. Lock mass option (m/z 445.1200025) from ambient air was enabled for internal calibration.

#### MS data analysis

Raw data from each replicate obtained from TgCDPK7 mutant parasites were searched against *T*. *gondii* (downloaded from ToxoDB web resource). Human proteins were included as contaminants. The search was carried out using Sequest and Mascot (version 2.4.1) search engines on Proteome Discoverer version 2.1 suite. A precursor and fragment ion mass tolerance of 10 ppm and 0.05 Da was used. Phosphorylation of serine, threonine, tyrosine and oxidation of methionine were selected as dynamic modifications. Carbamidomethylation of cysteine residues and TMT labeling at peptide N-terminus and lysine residues were selected as static modifications. Percolator node was used to compute false discovery rate (FDR) at PSM level and posterior error probability score. The data was filtered at 1% PSM level FDR and normalized against the total protein abundance as mentioned earlier [6]. We used ptmRS node to calculate probability of phosphorylation site localization and used a cut-off of > 75% ptm RS score to identify phosphorylation sites.

List of phosphosites and their respective expression fold change (mutant/control) across biological replicates were uploaded on Perseus software (version 1.4) to compute p-value for each phosphosite and generate S-curve [34]. Student t-test was used to assign p-values to each phosphosite based on its phosphorylation fold-change in mutant parasites across biological replicates and phosphosites with p-value<0.05 were considered for downstream analysis. We further applied a minimal cut-off of 1.33-fold change for differentially phosphorylated sites based on our observations in our previous study [6]. Pathway and gene ontology analysis of differentially phosphorylated *T*. *gondii* proteins was carried out using gene ontology tools available on ToxoDB web resource. Protein-protein interactions were predicted as described earlier [6]. In brief, list of differentially phosphorylated proteins was loaded on STRING web resource (version 10.0) and interactions were predicted using medium level confidence [35]. Proteins that were not predicted to interact with any other protein were removed from the representation and protein interactions under a common biological process were clustered together manually.

### *Toxoplasma* cultures, plaque and growth rate assays

*Toxoplasma gondii* tachyzoites RHΔhxgprt [36] and RHΔhxgprtΔKu80 [37] were grown in human foreskin fibroblast HFFs and maintained in Dulbecco’s modified Eagle’s medium supplemented with 10% fetal calf serum and 2mM glutamine at 37°C, 5% CO_2_ in humidified incubator. Protein knockdown in Tet-off system was mediated by addition 1μg/ml anhydrotetracyclin (ATc).

### Generation of transgenic parasite lines

#### TgCDPK7-iKD/ TgCDPK7-3HA

A region of genomic fragment of 2041 bp corresponding to the N-terminal coding sequence using primer pair 123/93 of *TgCDPK7* gene was amplified by PCR and subcloned into *Avr*II and *Spe*I along with the Kozak sequence in TATi-HXGPRT-tetO7Sag1 vector [38]. The 5′ flanking region of the TgCDPK7 promoter (1800 bp) was amplified by genomic PCR using primer pair 90/91 and cloned into the *Nco*I and *Bam*HI sites in TATi-HXGPRT-tetO7Sag1-NtCDPK7 construct to yield pCDPK7-TATi-HXGPRT-tetO7Sag1-NtCDPK7. ΔKu80 parasites were transfected using electroporation with 40 μg of this vector (linearized with *Nco*I and *Sbf*I) and subjected to MPA and xanthine selection. Successfully integrated inducible knock out parasites were subsequently subjected to limiting dilution cloning. To confirm integration and subsequent modification of the *TgCDPK7* locus, a series of PCRs were performed on the genomic DNA isolated from the selected clones. Two independent positive clones were obtained and most studies were performed on one of these and some key experiments were also repeated on the other clone. Most studies were carried out using this TgCDPK7-iKO line in which CDPK7 was not tagged. Subsequently, TgCDPK7 was tagged with 3xHA at C-terminus in TgCDPK7-iKD parasites (TgCDPK7-HA-iKD) or ΔKu80 (TgCDPK7-HA) with pLIC_DHFR vector using a strategy described previously [8] and selected with 1μM pyrimethamine.

#### Depletion of TgCDPK7

Typically, for knockdown of TgCDPK7, TgCDPK7-iKD or related parasites were pretreated with 1μg/ml ATc for 48h followed by additional incubation with ATc for another 24h (total treatment 72h) unless stated otherwise.

#### DDmycTgRab11a or DDmycTgRab11b overexpression in ΔKu80 or TgCDPK7-iKD parasites

TgRab11a was overexpressed with N-terminal ddFKBP and myc-tagged TgRab11a using a tubulin promoter. For this purpose, *TgRab11a* (660bp) was PCR amplified from *T*. *gondii* cDNA using primer Rab11afDDFH2/Rab11arDDFH2 and cloned in pGEMT-TA vector. TgRab11a S207/T205 was mutated to alanine (S207A/T205A) or aspartic acid (S207D) using site directed mutagenesis. Subsequently, TgRab11a wild type and S207A/D or T205A mutant were cloned in pTubDDFH2_CAT plasmid using *Nsi*I and *Pac*I restriction sites. These plasmids were transfected in ΔKu80 or TgCDPK7-iKD or TgCDPK7-HA-iKD (Figs 3D, 6D, S4 and S14A) or TgCDPK7-HA (Fig 3C, 6C and S14C) parasites and were selected using chloramphenicol for 20 days.

For Rab11b overexpression, TgRab11b cDNA was amplified from cDNA using primer Rab11bfDDFH2/Rab11brDDFH2 and cloned in pTubDDFH2_CAT plasmid using *Nsi*I and *Pac*I restriction sites. These plasmids were transfected in TgCDPK7-iKD (S9B Fig).

#### TgGPAT-Myc/Ty tagged in TgCDPK7-HA and TgCDPK7-iKD

A Myc or Ty tag was introduced in endogenous TgGPAT after amino acid residue 256 using CRISPR/Cas9 [39]. A gRNA specific to *TgGPAT* was cloned in pSAG1-Cas9GFP:UPRT plasmid using site directed mutagenesis and primer pair gRNAF/4883rev. ΔKu80 or TgCDPK7-iKD parasites were co-transfected with TgGPAT specific annealed oligonucleotides with 5’ and 3’-homology arm flanking the myc-tag and 50 μg of circular pSAG1-Cas9GFP:TgGPAT plasmid and TgCDPK7 C-terminal HA tagging construct (mentioned above). Parasites were selected using 1μM pyrimethamine for 3 cycles.

#### TgGPAT-Ty/myc overexpression in ΔKu80 or TgCDPK7-iKD parasites

TgGPAT (TGGT1_256980) cDNA 3003bp gene was synthesized from GeneArt (ThermoFisher Scientific) with EcoRI and NsiI restriction sites. TgGPAT cDNA was subcloned in pT8-Ty or myc_Bleo vector in EcoRI/NsiI sites for overexpression as C-terminal Ty or myc tag fusion protein. S244A and S278A mutations were introduced using site-directed mutagenesis sequentially and were subsequently confirmed by DNA sequencing. For transient overexpression, 100 μg TgGPAT or S244A/S278A double mutant plasmid was electroporated in ΔKu80 parasites and parasites were fixed after 48 hours of intracellular growth for immunofluorescence assay.

### Plaque and intracellular growth rate assay

For plaque assays, HFF monolayer was grown in 6 well plates and was infected with extracellular tachyzoites and washed with media after 2 hours. Cells were kept undisturbed at 37°C, 5% CO_2_ in humidified incubator for 10 or 15 days with or without 1μg/ml ATc and 200μM ethanolamine. Monolayer was washed and fixed with 4% PFA for 20 minutes and stained with Giemsa solution for 30 minutes. After three washings with PBS buffer, plaques were visualized and scanned under a light microscope.

Parasites pretreated with or without 1μg/ml ATc and cultured in the absence or presence of 200μM ethanolamine or choline for 48 hours were allowed to invade host cells for 2 hours. After 24 hours, cells were fixed with 4% PFA for 20 minutes and stained using anti-GAP45 antibody. Number of parasites per vacuole was determined and around 200 vacuoles were counted for each condition.

Almost similar protocol was followed for these assays using parasites overexpressing DDmycTgRab11a or its S207A mutant except that parasites were treated with 1μM Shld-1 for 16h. For invasion assays, naturally egressed parasites were incubated with fresh HFFs for additional 1h.

### Immunoprecipitation

For immunoprecipitation, TgCDPK7 was tagged with 3xHA at C-terminus, endogenous TgGPAT was tagged with Myc or DDmycTgRab11a was overexpressed in TgCDPK7-3HA tagged parasite line as described above. Parasites were cultured for 48 h, harvested and lysed by ultrasonication in RIPA buffer containing. Cell lysate was incubated with mouse anti-myc tag or anti-HA antibody for 12h, followed by incubation at 4°C for 6 h with Protein A/G agarose. Subsequently, the resin was washed with RIPA buffer and proteins bound to the resin were solubilized in 1× SDS-loading dye buffer and boiled for 10 min. Supernatant containing immunoprecipitated proteins was collected after centrifugation at 800g for 5 minutes followed by SDS-PAGE. Western blotting was performed using anti-HA or anti-myc antibody.

### Subcellular fractionation

Parasites were harvested at 48h post infection, centrifuged at 80 × g for 10 min to remove host cell debris, parasites were collected by further centrifugation at 800 × g for 10 min. The parasite pellet was lysed using freeze thaw cycles and was syringe lysed twice in cold phosphate-buffered saline (PBS) followed by solubilization of the pellet in 0.1M Na_2_Co_3_/PBS pH 11.2 followed by solubilization in 1% Triton X-100/PBS. For each round of solubilization, centrifugation was performed at 13000 rpm for 5 minutes at 4°C. Pellet and soluble fractions were subjected to SDS-PAGE gel electrophoresis and Western blotting was performed using Ty antibody.

### Real time PCR

Parasites were treated with or without 1μg/ml ATc for 72 hours and harvested for RNA isolation using Qiagen RNA isolation kit. Quantitative real-time PCR was performed in a Master cycler RealPlex machine (Eppendorf) using primer sets specific for TgCDPK7 or CDPK3 and Actin, which were used as control. The amplification reaction mixture contained 100ng template cDNA, 2× DyNAmo Color Flashq PCR master mix (Thermo Scientific) and 200 nM gene-specific primers.

### Immunofluorescence assays

HFF cells were cultured onto glass coverslips in 12 well plates. Parasites were allowed to invade for 2 hours and washed thrice subsequently and grown for 24 hours before fixation with 4% PFA in phosphate-buffered saline (PBS) followed by 0.2% Triton X-100 and blocked with 3% bovine serum albumin (BSA) for 30 min at room temperature. The parasites were incubated with indicated primary antibodies at 4°C, washed with PBS and incubated with Alexa Fluor 488 or 594 (488/594)-labeled secondary antibodies (Invitrogen) at room temperature. Following washing, mounting medium containing 4′,6′-diamidino-2-phenylindole (DAPI) was used to stain the nucleus. The stained parasites were visualized using Axio Imager Z1 microscope or LSM700 confocal microscope (Carl Zeiss). Z stacking during image acquisition and processing of images was done using AxioVision 4.8.2 software. Z-stacks that best represented the immunolocalization were used for illustrations in the figures. Adobe Photoshop software was also used for preparing images for figures.

### Western blotting

Freshly egressed *T*. *gondii* parasites were used to prepare lysates in a buffer containing 10 mM Tris (pH 7.5), 100 mM NaCl, 5 mM EDTA, 1% Triton X-100, and Complete protease inhibitor mixture (Roche Applied Science) protease inhibitor cocktail. After protein estimation and SDS-PAGE proteins were transferred to nitrocellulose membranes. Immunoblotting was performed using various primary antibodies and antisera, and HRP-labeled anti-rabbit IgG. WestPico or West Dura enhanced chemiluminescence (ECL) substrate (Pierce) was used to develop blots following manufacturer’s instructions.

### Lipid analysis and mass spectrometry-based lipidomic analyses

*T*. *gondii* intracellular tachyzoites (3×10^8^ cell equivalents) were harvested as described above for proteomics studies. Their total lipid spiked with 20 nmol C21:0 phosphatidylcholine was extracted by chloroform:methanol, 1:2 (v/v) and chloroform:methanol, 2:1 (v/v). The pooled organic phase was subjected to biphasic separation by adding 0.1% KCl and was then dried under N2 gas flux prior to being dissolved in 1-butanol. For the total fatty acid analysis, an aliquot of the lipid extract was derivatized on-line using MethPrepII (Alltech) and the resulting FA methyl esters were analyzed by GC-MS as previously described [40]. For the quantification of each lipid, total lipid was separated by 2D HPTLC using chloroform/methanol/28% NH4OH, 60:35:8 (v/v/v) as the 1st dimension solvent system and chloroform/acetone/methanol/acetic acid/water, 50:20:10:13:5 (v/v/v/v/v) as the 2nd dimension solvent system [41]. Each lipid spot was extracted for quantification of fatty acids by gas chromatography-mass spectrometry (Agilent 5977A-7890B) after methanolysis [40]. Fatty acid methyl esters were identified by their mass spectrum and retention time, and quantified by Mass Hunter Quantification Software (Agilent) and the calibration curve generated with fatty acid methyl esters standards mix (Sigma CRM47885). Then each lipid content was normalized according to the parasite cell number and a C21:0 internal standard (Avanti Polar lipids). All analyses were performed in triplicate or more (n = 3). The lipids were normalized with respect to number of parasites and reported relative to total FA. P values of ≤ 0.05 from statistical analyses (t-tests) obtained from GraphPad software analysis were considered statistically significant.

### Labeling of PL in *T*. *gondii*

Parasites were left untreated or pretreated with ATc for 48 h and grown for 24 h further in the presence or absence of ATc. Intracellular parasites were released using a syringe and washed with PBS thrice followed by removal of host cell debris by centrifugation (300 × *g*, 5 min). Parasites in the supernatant were pelleted (2000 × *g*, 10 min) and washed twice with complete DMEM medium. Tachyzoites (3×10^9^) were incubated with [1,2-^14^C] ethanolamine (20–40 nCi/nmol) or [1,2-^14^C] choline (0.1 nCi/nmol) in 1 ml of culture medium in glass tubes (8 h, 37°C) followed by lipid extraction as described previously [42].

In some experiments that were aimed at assessing if host derived PLs are used by the parasites, HFF cells grown in T25 flasks were labeled with [1,2-^14^C] ethanolamine (5 μCi, 25 μm in DMEM) for 2 days and were then infected with tachyzoites which were supplemented with a 200-fold excess of unlabeled ethanolamine (5mM) to dilute the residual pool of radioactive ethanolamine in the host cells. The intracellular parasites were further grown for 48 hours before they were released. Extracellular parasites were washed twice and subjected to lipid extraction.

### Lipid extraction and analysis

Total lipid isolation from *T*.*gondii* was performed using a previously described method [42]. Normalized parasites pellets were suspended in PBS and were mixed after addition of chloroform and methanol. Lipids were recovered from organic phase after centrifugation and dried under nitrogen gas stream and suspended in chloroform and methanol (9:1). Lipids were resolved on TLC on silica gel-60 plates in chloroform, methanol, and NH_4_OH (65:25:4, v/v/v) and visualized by phosphorimaging. The fold change in radiolabeled PLs was determined after densitometry quantitation of radiolabelled bands/spots, which was done using Image J software.

### Expression of recombinant GST-ΔTgCDPK7

ΔTgCDPK7 (PH along with kinase domain,a.a 1426–1780) was PCR amplified and cloned in *Bam*HI and *Not*I restriction sites of the pGEX4T1 vector for recombinant protein expression. Recombinant proteins were expressed in *Escherichia coli* BL-21 strain. The cultures were grown at 37°C until an optical density at 600 nm of 0.6 to 0.8 was reached and induced with 1 mM isopropyl-β-d-thiogalactopyranoside (IPTG) at 18°C for 16 h. Cells were pelleted by centrifugation at 4,000 rpm for 10 min at 4°C and suspended in 50 mM Tris-HCl (pH 7.4), 2 mM EDTA, 1% Triton X-100, 1 mM dithiothreitol (DTT), and protease inhibitors. Samples were sonicated for 10 cycles, and supernatant was used to affinity purify recombinant protein using glutathione sepharose.

cDNA for TgRab11a or its S207A mutant was subcloned in pET28a+ vector using *Bam*HI and *Not*I. Recombinant protein was expressed in BL21 DE3 cells and purified by affinity chromatography performed using Ni-NTA agarose.

A region of TgGPAT (aa. 205–413) that codes for a protein which has S244 and S278 was amplified from cDNA and cloned in pGEX4T-1 vector in *Bam*HI and *Not*I sites. Recombinant protein was expressed in BL21 cells by affinity chromatography using glutathione sepharose.

### Kinase assay

Kinase assays were performed by incubating recombinant proteins 2 μg His-TgRab11a or 5.6 μg GST-TgGPAT (aa 205–413) as substrates with 5 μg recombinant TgCDPK7 kinase domain protein in a kinase assay buffer (50mM Tris pH 7.5, 10mM MgCl_2_, 1mM DTT and 100 μM ATP) with [γ-^32^P Adenosine Tri-Phosphate] (15 μCi/reaction) for 40 minutes at 30°C. The reaction was stopped by boiling assay mix in SDS-loading buffer for 10 minutes. Proteins were separated on SDS-PAGE and phosphorylation of proteins was detected by phosphorimaging using a FUJI FLA5000 or GE STORM scanner.

## Supporting information

S1 Text Supplementary Methods. (PDF)

S1 Data Phosphoproteomics related dataset. **1.1:** List of phosphopeptides identified in the phosphoproteomic analysis of TgCDPK7 parasites. **1.2:** List of hyposphosphorylated sites identified upon depletion of TgCDPK7. **1.3:** List of hypersphosphorylated sites identified upon depletion of TgCDPK7. **1.4:** Pathways enriched in hypophosphorylated T. gondii proteins. **1.5:** Biological processes enriched in hypophosphorylated T. gondii proteins. **1.6:** Protein-protein interaction analysis on the differentially phosphorylated T. gondii proteins. **1.7:** TMT labeling details for the quantitative phosphoproteomic analysis of TgCDPK7 parasites.
(XLSX)

S1 Table PCR Primers. (PDF)

S1 Fig A. Schematic representation of the strategy used to generate TgCDPK7-iKD parasites. A plasmid construct that allowed insertion of transactivator TATi-1 and replacement of *TgCDPK7* promoter with the 7tet-Op SAG1 (TetO7) inducible promoter was introduced by double homologous recombination in TgCDPK7 locus. After transfection, the drug selected parasites were cloned by limiting dilution. B. Genotyping of TgCDPK7-iKD parasites. Although two independent clones were obtained, clone#1 (TgCDPK7-iKD) was used for most reported studies and some experiments were performed with clone #2 (Panel E). PCR amplification of the unmodified and recombined locus was performed using indicated primers (Panel A, S1 Table) for confirming 5’- and 3’- integration. PCR products of expected size were obtained and the wild type (WT) locus was absent in the transgenic TgCDPK7-iKD parasites. C. TgCDPK7-iKD parasites grown in the absence or presence of ATc for 72h. Real time PCR was performed for assessing the expression of TgCDPK7. TgCDPK3 was used as a control with respect to which TgCDPK7 expression was determined (Mean+/-SE, *n = 3, p<0.0001, t-test). D. ΔKu80 parasites were pre-incubated for 48h with ATc and were subsequently allowed to invade fresh HFFs in the presence or absence of ATc. The number of parasites per vacuole was determined after 24h. ATc treatment did not alter replication of ΔKu80 parasites. There was no significant difference in parasite growth upon ATc treatment. E. TgCDPK7-iKD_clone 2 was used for performing parasite replication assays in the presence or absence of ATc and ethanolamine as described in Fig 5D. (mean±SE, *n = 3, p<0.01 for 8 parasites/vacuole, ANOVA).
(PDF)

S2 Fig A. Plaque assays were carried out by infecting HFF monolayer with ΔKu80 or TgCDPK7-iKD in the presence or absence of ATc with or without 200 μM Eth or 200 μM choline after 10 and 15 days post treatment, respectively and number of plaques were counted after the treatment (B).
(PDF)

S3 Fig A-B. Recombinant TgGPAT (aa. 205–413) (Lane1, Panel A) and a N-terminal deletion mutant that only has the PH and the kinase domain of TgCDPK7 (ΔTgCDPK7) (Lane 2, Panel A) were expressed as GST-fusion proteins. TgRab11a (B) was expressed as 6xHis tagged protein. All proteins were purified by affinity chromatography. A SDS-PAGE gel of the purified recombinant proteins, which were used for kinase assays, is shown here.
(PDF)

S4 Fig TgCDPK7-HA-iKD parasites were cultured in the presence or absence of ATc for 48h. Subsequently, parasites were harvested followed by Western blotting using anti-HA antibody to detect TgCDPK7-HA. *-a possible breakdown/spliced product. Actin was used as a loading control. *Right panel*, Densitometry of TgCDPK7-HA band in the Western blot was performed to determine the fold change in its expression upon ATc addition.
(PDF)

S5 Fig A-C.TgCDPK7-iKD parasites were treated with ATc for 72h following which IFA was performed using antibodies against apicoplast protein Cpn60 (A), microneme protein MIC2 (B), rhoptry protein ROP2 (C) along with GAP45. Microneme (B) and rhoptry (C) proteins were mislocalized upon ATc treatment. D. IFA of TgCDPK7-iKD parasites revealed the presence of IMC1 on newly formed IMCs in the absence of ATc. In ATc-treated parasites, IMC was either missing or disorganized (arrows). E. GRASP-YFP [43] was transiently overexpressed in TgCDPK7-iKD parasites followed by ATc treatment for 72h. ATc treatment resulted in either aberrant (arrows) or missing/diffuse (*) Golgi. F. IFA performed on untreated or ATc-treated parasites using anti-centrin1 antibody revealed that centrosomes were present either in abnormal numbers and/or unusual location (arrows) or were absent (*) upon ATc treatment.
(PDF)

S6 Fig **A-E.** DDmycTgRab11a or S207A parasites were grown in the presence or absence of Shld-1 for 4h. GRASP-YFP was transiently transfected to detect Golgi (A) or antibodies against centrin 1 (B), IMC1 (C) and anti-GRA3 (D), MIC2 (E) were used to detect centrosomes, micronemes and dense granules, respectively. IFA revealed abnormal number/location (arrows) or missing/diffuse (*) Golgi in S207A parasites (A). Centrosomes were stretched (arrow) or diffuse/missing (*) in the S207A mutant parasites (B). IMCs were not observed or was diffused (*) or not in sync (arrow) in S207A mutant (C). Dense granules (D) and microneme (E) were almost unaltered in mutant parasites. F. GPAT-myc was transiently transfected in DDmycTgRab11a or S207A parasites, which were treated with Shld-1. IFA with anti-myc antibody revelaed perinuclear ER-like staining for GPAT in both WT TgRab11a and S207A parasites.
(PDF)

S7 Fig DDmycTgRab11a or S207A parasites were grown in the presence or absence of Shld-1 for 16h. Subsequently, invasion assays were performed and infected host cells were determined. There was no significant difference in invasion by Rab11a or S207A overexpressing parasites (ANOVA, n = 2, ns-not significant).
(PDF)

S8 Fig A. IFA was performed on DDmycTgRab11a-T205A or DDmycTgRab11a-S207D overexpressing parasites in presence of Shld-1 for 4h as described in Fig 4D using antibodies against SAG1 and GAP45. B. IFA was performed for SAG1 and GAP45 on DDmycTgRab11a or S207A/D parasites in presence of Shld-1 for 4h as described above. Eth was added for 120h prior to addition of Shld-1 to one set of S207A parasites. Eth supplementation did not reverse the mislocalization of SAG1 in S207A parasites.
(PDF)

S9 Fig A. TgCDPK7-iKD/DDmycTgRab11a parasites were treated with ATc in the presence of Shld-1. Subsequently, parasite pellets were isolated and used for extracting proteins in PBS, Na_2_CO_3_ pH 11.0 or 0.5% Triton X-100. Western blotting using anti-myc antibody was performed on various fractions. B. TgCDPK7-iKD/DDmycTgRab11b in which TgRab11b was expressed in the presence of Shld-1 as N-terminal DD myc tag fusion protein in TgCDPK7-iKD background was treated with ATc followed by IFA using anti-myc antibody for detecting TgRab11b and anti-GAP45. C. TgCDPK7-HA/DDmycTgRab11a WT or S207A parasites were treated with Shld-1 for 4h. Subsequently, IFA was performed using anti-HA (to detect TgCDPK7-HA) and SAG1 antibodies. There was no significant difference in TgCDPK7 localization whereas SAG1 was mislocalized in TgRab11a S207A parasites as described in the main text.
(PDF)

S10 Fig Protein-protein interactions were predicted between differentially phosphorylated proteins in TgCDPK7-iKD proteins upon ATc addition (Fig 2B and S1 Data, 1.5) using STRING resource. The analysis exhibited high confidence protein-protein interactions between the candidate genes. The modules involving TgGPAT and TgRab11a discussed in Fig 2D and 2E are encircled.
(PDF)

S11 Fig Western blotting using anti-SAG1/ROM4 antibody showed unaltered protein expression upon TgCDPK7 depletion with ATc for 72 hours.
(PDF)

S12 Fig PLs were extracted from TgCDPK7-iKD tachyzoites grown in the presence or absence of ATc as described in Panel A. Relative abundance of individual molecular species in total FA (A), which shows the significant decrease of C16:0, C18:1 Trans and the significant increase in C20:1, C20:4 and C20:5 upon TgCDPK7 depletion. FA profiles were also quantified for the phospholipid classes PE (B) and PC (C) (mean±, SE, *n = 3, p<0.05, t-test (B,C)/ANOVA(A)).
(PDF)

S13 Fig A. Metabolic labeling to monitor the synthesis of PC and PE via the parasite Kennedy pathway in parasites overexpressing either WT TgRab11a or its S207A mutant as described earlier in Fig 5B. Extracellular tachyzoites were incubated with ^14^C-Eth or ^14^C-Cho in the presence or absence of Shield-1 (16h treatment). Subsequently, lipids were extracted and radiolabeled lipids were detected by phosphorimaging of TLC. There was no significant difference in PC or PE levels. *Right Panel*, Densitometry of spots corresponding to PC/PE was performed and fold change in PC/PE in S207A mutant with respect to wild type TgRab11a was determined (Mean ± SE, ANOVA, n = 2, ns-not significant). B. WT TgRab11a or its S207A mutant parasites were incubated with or without 200 μM ethanolamine in the presence of Shield-1 for 16 hours. The number of parasites per vacuole was determined after 24h. Data represent Mean ± SE, n = 3 and at least 200 vacuoles were counted for each condition.
(PDF)

S14 Fig A. GPAT was myc-tagged at endogenous locus in its linker region as described in Fig 6A in TgCDPK7-iKD parasites (TgCDPK7-iKD/GPAT-myc). ATc was added to deplete TgCDPK7 followed by IFA for GPAT-myc as described in Fig 6D. There was a significant change in localization of GPAT from perinuclear ER like compartment (-ATc) to predominantly parasite cytoplasm upon TgCDPK7 depletion (+ATc). B. GPAT-myc/TgCDPK7-iKD parasites were treated with ATc for 72h followed by Western blotting with anti-myc antibody to detect GPAT-Myc. There was no significant change in GPAT expression upon ATc addition. C. TgCDPK7-HA/GPAT-Myc or TgCDPK7-HA/DDMyc-Rab11a parasites were cultured and parasite lysates were prepared from these or parental (ΔKu80) parasites and were used for IP with anti-myc. Subsequently, Western blotting was performed on GPAT-myc IP and DDmyc-Rab11a-IP using anti-HA antibody to detect TgCDPK7-HA. A band corresponding to TgCDPK7-HA was observed only in IP from transgenic parasites. No band was observed in the case of ΔKu80 line. D. TgCDPK7-HA/DDMyc-TgRab11a parasites were grown in presence of Shield-1 for 4h. IFA was performed using anti-myc and anti-HA antibodies. There were a few TgCDPK7 puncta that co-localized with TgRab11a-puncta.
(PDF)

S15 Fig TgCDPK7-iKD parasites were preincubated for 48h with ATc and were subsequently allowed to invade fresh HFFs in the presence or absence of ATc for 24h. In one case, 200 μM of Eth was added for 120h to cultures prior to addition of ATc. Subsequently, IFA was performed for SAG1 and ROM4. The addition of Eth caused a partial restoration of pellicle staining of SAG1. SAG1 was present at pellicle as well as vesicles but the staining was reduced in comparison to -ATc cells.
(PDF)
