# Supplementary material for: Protein kinase TgCDPK7 regulates vesicular trafficking and phospholipid synthesis in Toxoplasma gondii
Source: PLoS Pathog. 2021 Feb 26;17(2):e1009325. doi: 10.1371/journal.ppat.1009325 (PMC7909640; doi:10.1371/journal.ppat.1009325)
Supplement: S1 Table — (PDF) [file ppat.1009325.s003.pdf]

Supplemental Data Table S1: PCR Primers

Oligo	Forward (5'-3')	Reverse (5'-3')
LIC-HXGPRT-TgCDPK7-3HA	415TACTTCCAATCCAATTTAATGCAACACTCCGGTCTCTTTCGACGGCG	416 TCCTCCACTTCCAATTTTAGCCTTATGTACTTCCTCTATGCTAGGTGAGG
TgPH+KD(CDPK7)pGEX4T-1	101 GAC TAG GAT CCG CGG CCA TCG ACG GCG TTC	104 GTC AAG CGG CCG CCT AGA TCC AAA TGT GCT GGA GCG CA
5'UTR forTgCDPK7-iKD	90 GTA CGC CAT GGG AGA CAC ACG CAG ACT TAC TTC GTG	91 GTA CGG GAT CCC GAC CTT GGA GAC AGA GAA GAC
3'Coding sequence for TgCDPK7-iKD	123 TTCCTAGGGATATCAAAA TGG GGG GCG TCC AGT CGA CCC GCG	93 GTA GCA CTA GTG GAA GGG AGG ACG GAG ACT GGT CG
5'-integration for TgCDPK7-iKD	105 GTGGAAAATGCCAAGCAAATACTGCAG	106 CCTGAGACGCGTGTTCAAGCTTATC
3'-integration for TgCDPK7-iKD	107 CTGATAGGGAGTGGTAAACTCGAG	108 GCACTTGCGGCGGCG GAGACACTC
WT promoter TgCDPK7	137 GGTTTCTCCCGTCTGGCGAGGAAGGATTGTGC	138 CGCGGAGAAATCTGCGGACACGCACCCG
DDmycTgRab11a	Rab11afDDFH2 CCATGCATGCGGCTAAAGATGAATAC	Rab11arDDFH2 TCTTAATTAATCAGGCGGAACAGCAGCC
DDmycTgRab11a_S207A	Rab11afS207A AGCGAACAGATGCAC AAATACGGCAA	Rab11arS207A TTGCCGTATTTGTGCATCTGTTCGCT
DDmycTgRab11a_S207D	Rab11afS207D AGCGAACAGATG ACCAAATACGGCAA	Rab11arS207D TTGCCGTATTTGGT CATCTGTTCGCT

DDmycTgRab11a_T205A	Rab11afT205A<br>GAT GAG GAG CGA GCT GAT AGC CAA ATA	Rab11arT205A<br>TAT TTG GCT ATC AGC TCG CTC CTC ATC
DDmycTgRab11b	Rab11bfDDFH2<br>GCGATGCATGGGGGTTCTGAAGACTACG	Rab11brDDFH2<br>GCGTTAATTAACCACAGCAGGACAGATTCTGAGGG
PET28a- TgRab11a	TgRab11aF<br>CTGGATTCCGCGGCTAAAGATGAATACTACG	TgRab11aR<br>CTGCGGCCGCTCAGGC GGA ACA GCA GCC
pGEX4T1-TgGPAT	TgGPAT_FP<br>CGGGATCCACGAAGAACGAAGAAGATCCG	TgGPAT_RP<br>AAGCGGCCGCTCGAACCGCTACTACTCG
Real Time PCR TgCDPK7(182-292bp)	210<br>GTCCCGGACCCGGAATCGA	211<br>CACGCTTCCCGAGCCTTTG
Real Time PCR TgCDPK7(5615-5729bp)	AGTGAAGCCGCGATCGAC	CGCCCTTTCGCCGGTAGTA
Real Time PCR TgCDPK3(43-153bp)	208<br>GGCGCAGCTGGAGAAAAACC	209<br>GTGTCGGGAGACCCAGTTC
TgGPAT-myc oligos(endogenous tagging)	GPAT-Myc F<br>GCCAGAGCTGCGCGCGCACACACGAGGTGAATCGCCTGCGAAACTACTTGCCGGCGAGCAGAAGCTCATCTCCGAGGAGGACCTGGATGAGATGCTGCAGGCTTTTTCTCGAGTGCCAT	GPAT-Myc R<br>ATG GCA CTC GAG AAA AAG CCT GCA GCA TCT CAT CCA GGT CCT CCT CGG AGA TGA GCT TCT GCT CGC CGG CAA GTA GTT TCG CAG GCG ATT CAC CTC GTG TGT GCG CGC GCA GCT CTG GC
gRNA-GPAT	grnaF<br>ATCGCCCGCAAAGCTCCGTTTTAGAGCTAGAAATAGC	4883rev<br>AACTTGACATCCCCATTTAC
S244A TgGPAT	GPAT244Af<br>ACACGAGGTGAAGCGCCCGCAAAGCTC	GPAT244Ar<br>GAGCTTTGCGGGCGCTTCACCTCGTGT

S278A TgGPAT	GPAT278Af AGCAAGAGTGCAGCAGCGCCTCTCGGG	GPAT278Ar CCCGAGAGGCGCTGCTGCACTCTTGCT
TgGPAT myc or ty tagged overexpression	CAGAATTCCCTTTTTCGACAAAATGTATTTCCTTGTCCGGT	CAATGCATGGAGAATTTCCTCTGC
