# Supplementary figures and images for: Protein kinase TgCDPK7 regulates vesicular trafficking and phospholipid synthesis in Toxoplasma gondii
Source: PLoS Pathog. 2021 Feb 26;17(2):e1009325. doi: 10.1371/journal.ppat.1009325 (PMC7909640; doi:10.1371/journal.ppat.1009325)

**A**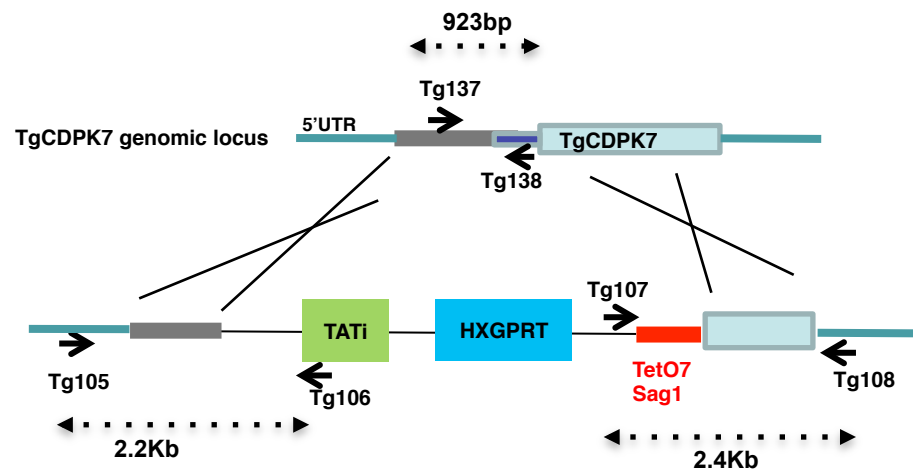**B**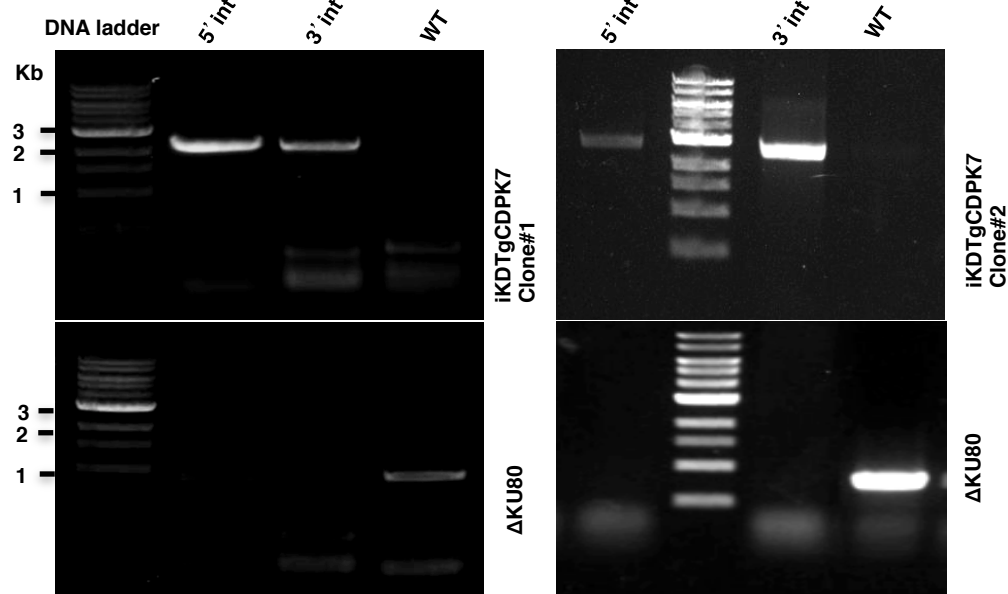**C**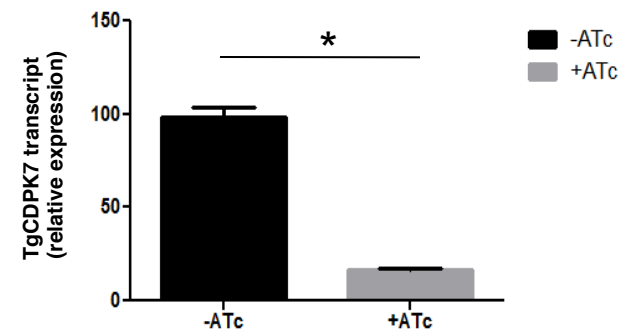**D**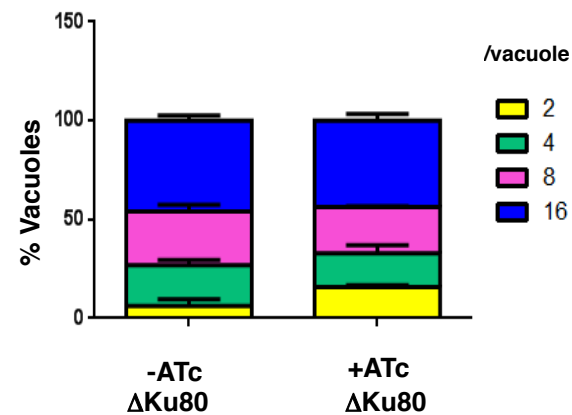**E**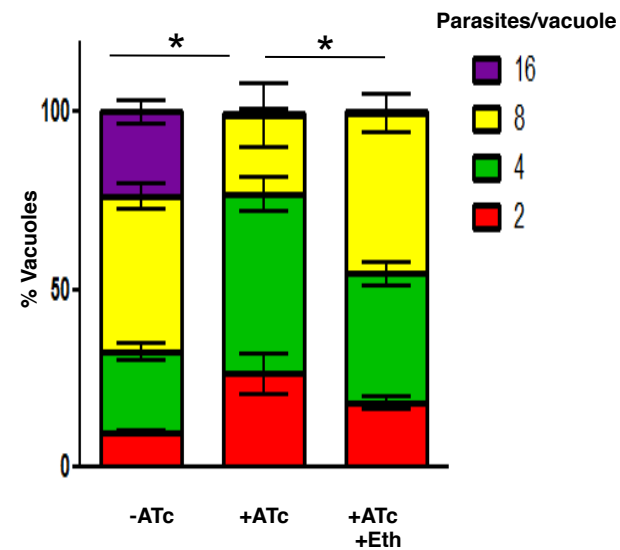

Supplement: S1 Fig — A. Schematic representation of the strategy used to generate TgCDPK7-iKD parasites. A plasmid construct that allowed insertion of transactivator TATi-1 and replacement of TgCDPK7 promoter with the 7tet-Op SAG1 (TetO7) inducible promoter was introduced by double homologous recombination in TgCDPK7 locus. After transfection, the drug selected parasites were cloned by limiting dilution. B. Genotyping of TgCDPK7-iKD parasites. Although two independent clones were obtained, clone#1 (TgCDPK7-iKD) was used for most reported studies and some experiments were performed with clone #2 (Panel E). PCR amplification of the unmodified and recombined locus was performed using indicated primers (Panel A, S1 Table) for confirming 5’- and 3’- integration. PCR products of expected size were obtained and the wild type (WT) locus was absent in the transgenic TgCDPK7-iKD parasites. C. TgCDPK7-iKD parasites grown in the absence or presence of ATc for 72h. Real time PCR was performed for assessing the expression of TgCDPK7. TgCDPK3 was used as a control with respect to which TgCDPK7 expression was determined (Mean+/-SE, *n = 3, p<0.0001, t-test). D. ΔKu80 parasites were pre-incubated for 48h with ATc and were subsequently allowed to invade fresh HFFs in the presence or absence of ATc. The number of parasites per vacuole was determined after 24h. ATc treatment did not alter replication of ΔKu80 parasites. There was no significant difference in parasite growth upon ATc treatment. E. TgCDPK7-iKD_clone 2 was used for performing parasite replication assays in the presence or absence of ATc and ethanolamine as described in Fig 5D. (mean±SE, *n = 3, p<0.01 for 8 parasites/vacuole, ANOVA). (PDF) [file ppat.1009325.s004.pdf]

A

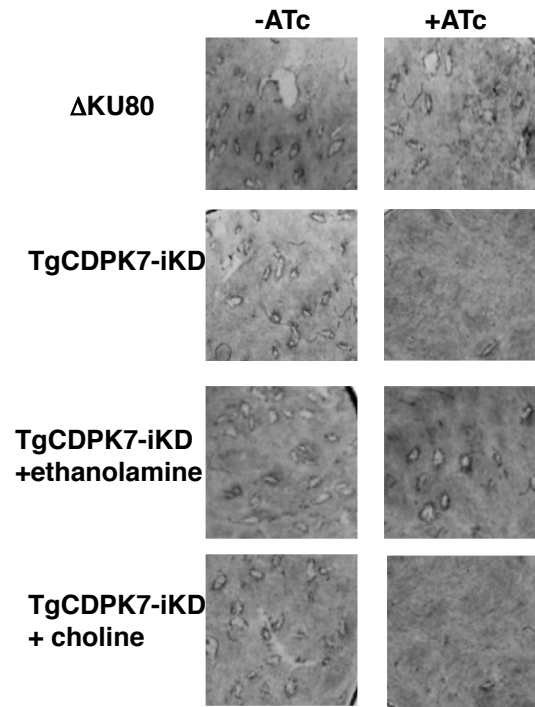

B

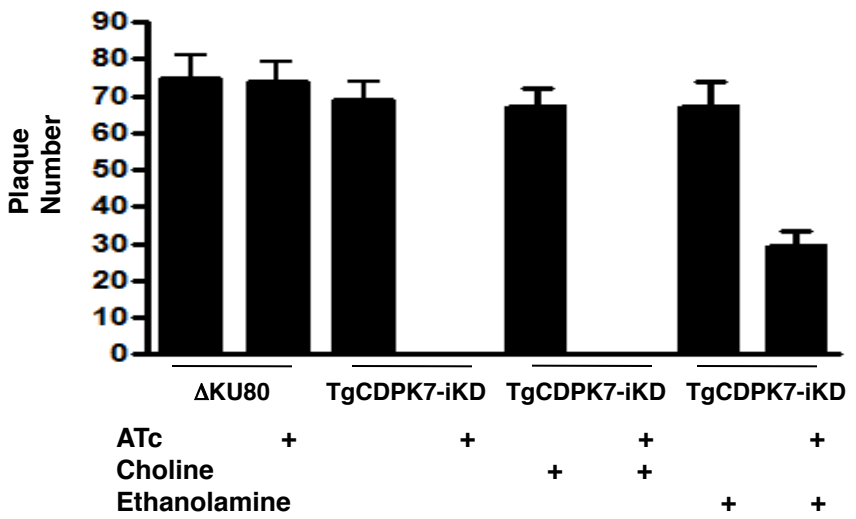

Supplement: S2 Fig — A. Plaque assays were carried out by infecting HFF monolayer with ΔKu80 or TgCDPK7-iKD in the presence or absence of ATc with or without 200 μM Eth or 200 μM choline after 10 and 15 days post treatment, respectively and number of plaques were counted after the treatment (B). (PDF) [file ppat.1009325.s005.pdf]

**A**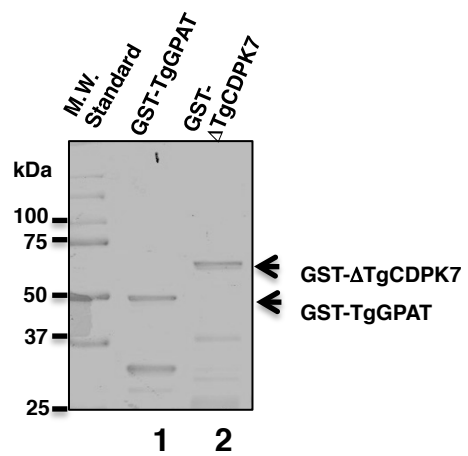**B**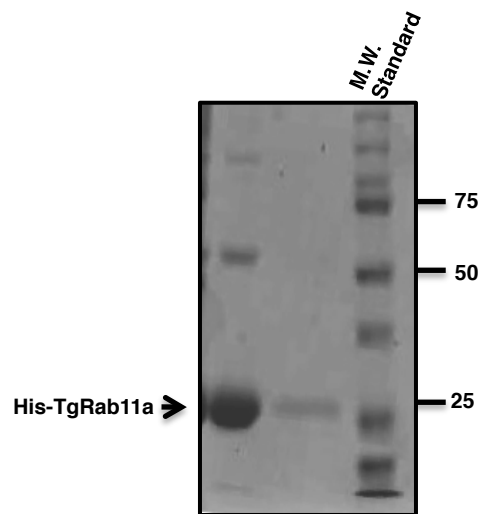

Supplement: S3 Fig — A-B. Recombinant TgGPAT (aa. 205–413) (Lane1, Panel A) and a N-terminal deletion mutant that only has the PH and the kinase domain of TgCDPK7 (ΔTgCDPK7) (Lane 2, Panel A) were expressed as GST-fusion proteins. TgRab11a (B) was expressed as 6xHis tagged protein. All proteins were purified by affinity chromatography. A SDS-PAGE gel of the purified recombinant proteins, which were used for kinase assays, is shown here. (PDF) [file ppat.1009325.s006.pdf]

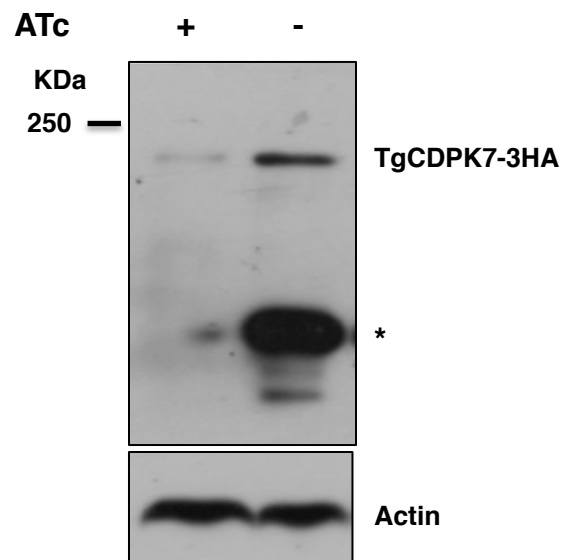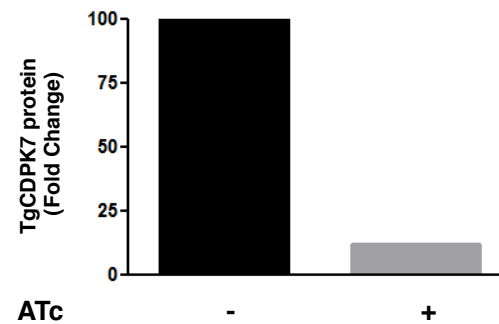

Supplement: S4 Fig — Subsequently, parasites were harvested followed by Western blotting using anti-HA antibody to detect TgCDPK7-HA. *-a possible breakdown/spliced product. Actin was used as a loading control. Right panel, Densitometry of TgCDPK7-HA band in the Western blot was performed to determine the fold change in its expression upon ATc addition. (PDF) [file ppat.1009325.s007.pdf]

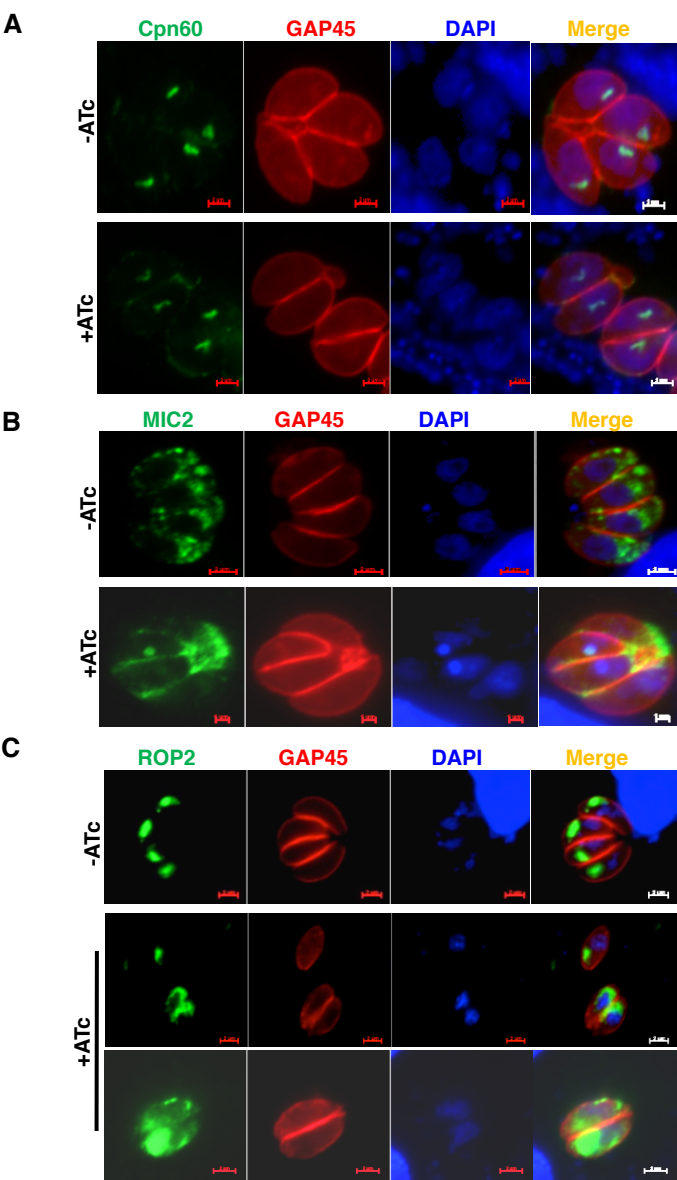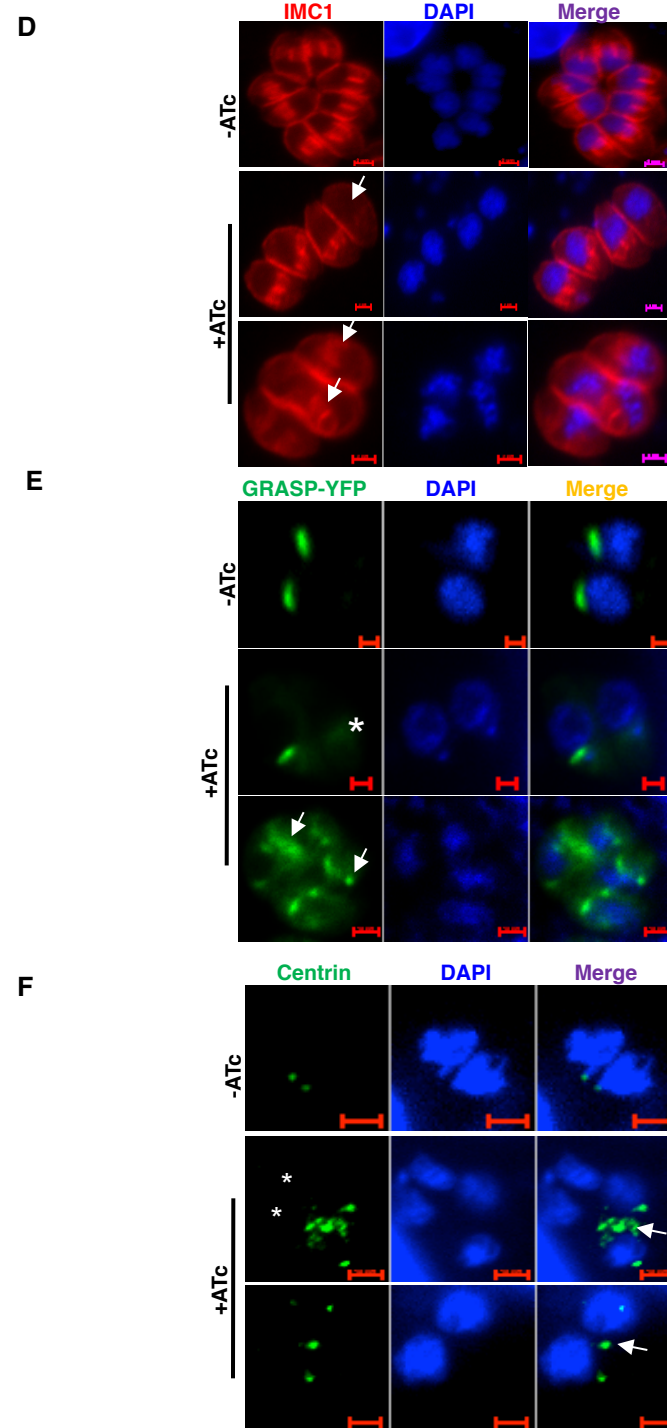

Supplement: S5 Fig — A-C.TgCDPK7-iKD parasites were treated with ATc for 72h following which IFA was performed using antibodies against apicoplast protein Cpn60 (A), microneme protein MIC2 (B), rhoptry protein ROP2 (C) along with GAP45. Microneme (B) and rhoptry (C) proteins were mislocalized upon ATc treatment. D. IFA of TgCDPK7-iKD parasites revealed the presence of IMC1 on newly formed IMCs in the absence of ATc. In ATc-treated parasites, IMC was either missing or disorganized (arrows). E. GRASP-YFP [43] was transiently overexpressed in TgCDPK7-iKD parasites followed by ATc treatment for 72h. ATc treatment resulted in either aberrant (arrows) or missing/diffuse (*) Golgi. F. IFA performed on untreated or ATc-treated parasites using anti-centrin1 antibody revealed that centrosomes were present either in abnormal numbers and/or unusual location (arrows) or were absent (*) upon ATc treatment. (PDF) [file ppat.1009325.s008.pdf]

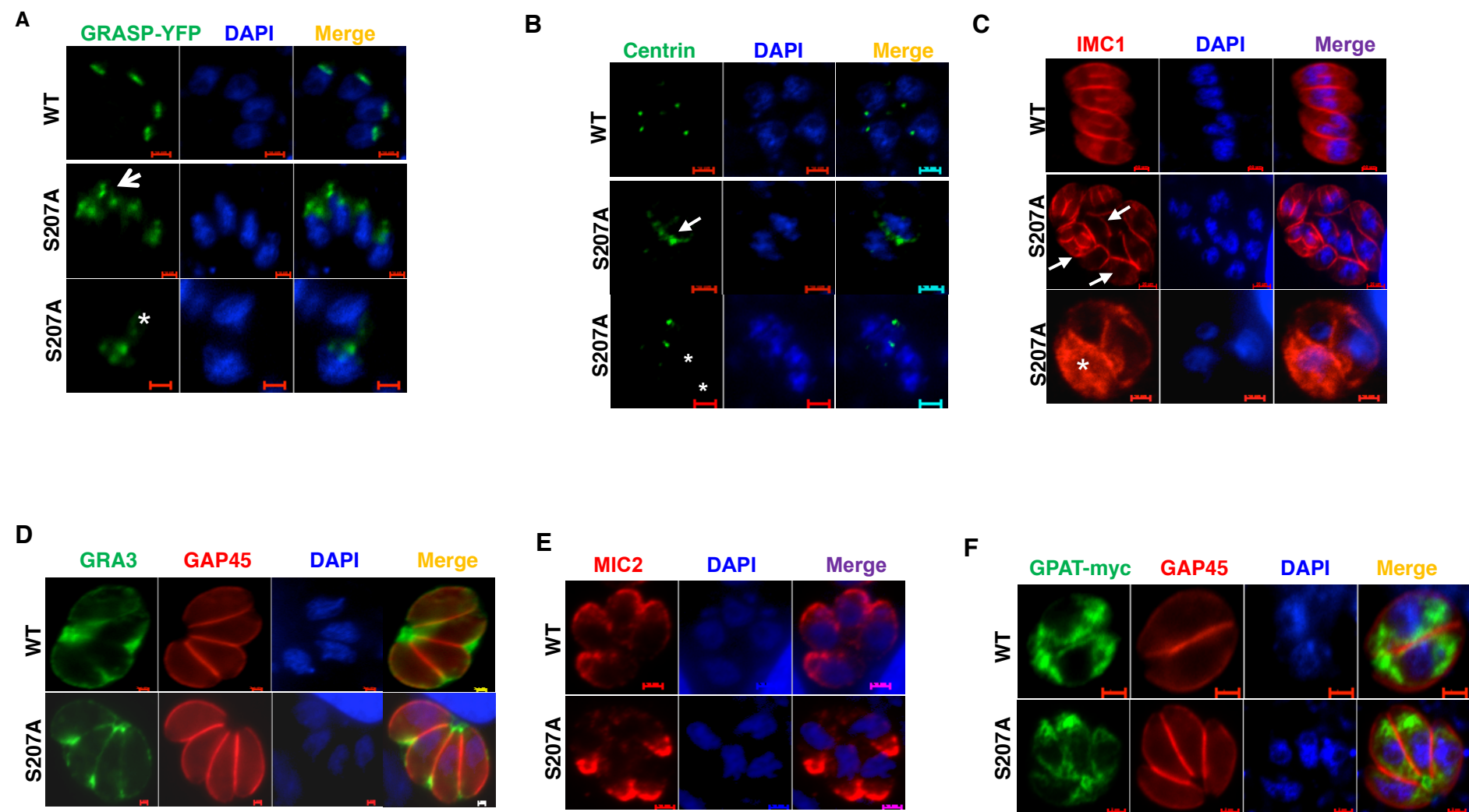

Supp. Fig. S6

Supplement: S6 Fig — A-E. DDmycTgRab11a or S207A parasites were grown in the presence or absence of Shld-1 for 4h. GRASP-YFP was transiently transfected to detect Golgi (A) or antibodies against centrin 1 (B), IMC1 (C) and anti-GRA3 (D), MIC2 (E) were used to detect centrosomes, micronemes and dense granules, respectively. IFA revealed abnormal number/location (arrows) or missing/diffuse (*) Golgi in S207A parasites (A). Centrosomes were stretched (arrow) or diffuse/missing (*) in the S207A mutant parasites (B). IMCs were not observed or was diffused (*) or not in sync (arrow) in S207A mutant (C). Dense granules (D) and microneme (E) were almost unaltered in mutant parasites. F. GPAT-myc was transiently transfected in DDmycTgRab11a or S207A parasites, which were treated with Shld-1. IFA with anti-myc antibody revelaed perinuclear ER-like staining for GPAT in both WT TgRab11a and S207A parasites. (PDF) [file ppat.1009325.s009.pdf]

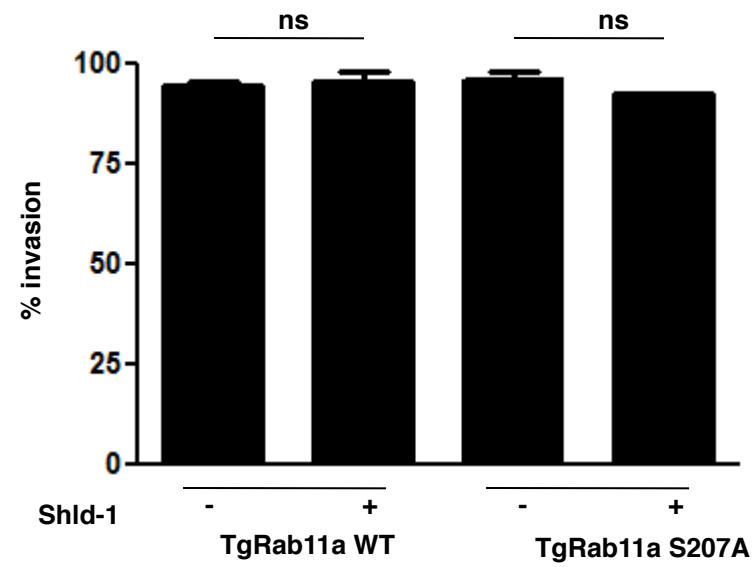

Supplement: S7 Fig — DDmycTgRab11a or S207A parasites were grown in the presence or absence of Shld-1 for 16h. Subsequently, invasion assays were performed and infected host cells were determined. There was no significant difference in invasion by Rab11a or S207A overexpressing parasites (ANOVA, n = 2, ns-not significant). (PDF) [file ppat.1009325.s010.pdf]

**A**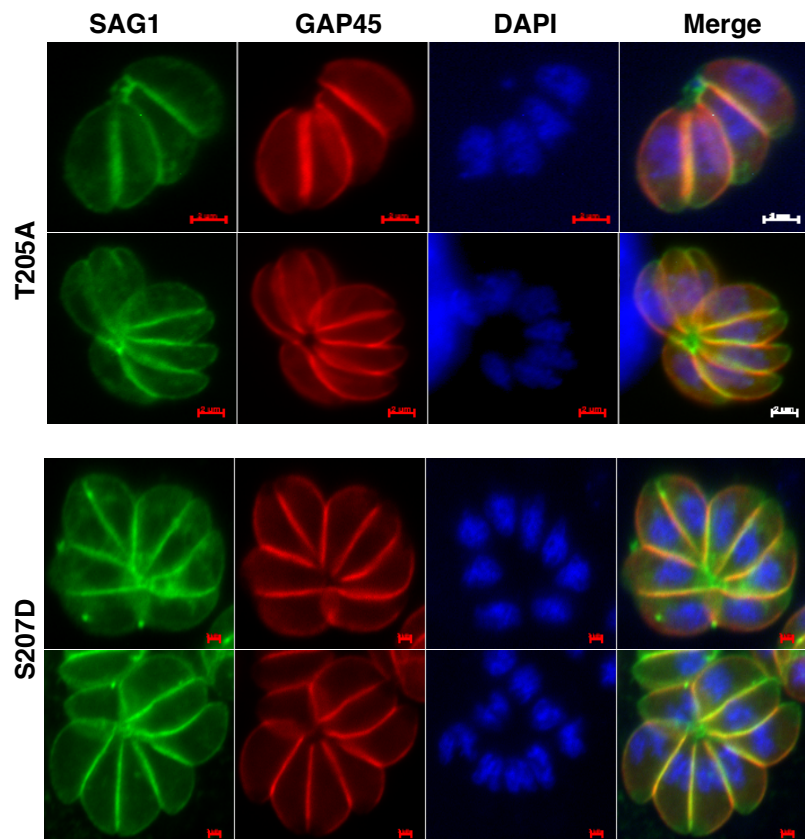**B**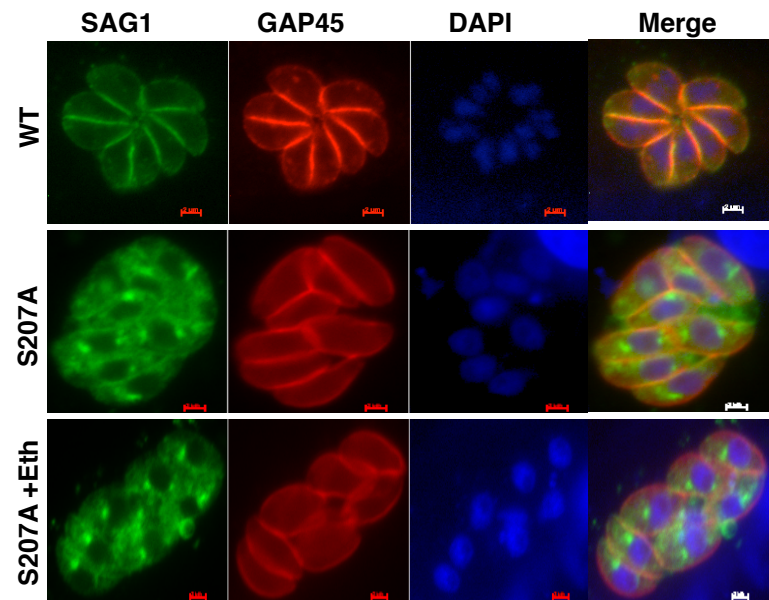

Supplement: S8 Fig — A. IFA was performed on DDmycTgRab11a-T205A or DDmycTgRab11a-S207D overexpressing parasites in presence of Shld-1 for 4h as described in Fig 4D using antibodies against SAG1 and GAP45. B. IFA was performed for SAG1 and GAP45 on DDmycTgRab11a or S207A/D parasites in presence of Shld-1 for 4h as described above. Eth was added for 120h prior to addition of Shld-1 to one set of S207A parasites. Eth supplementation did not reverse the mislocalization of SAG1 in S207A parasites. (PDF) [file ppat.1009325.s011.pdf]

**A**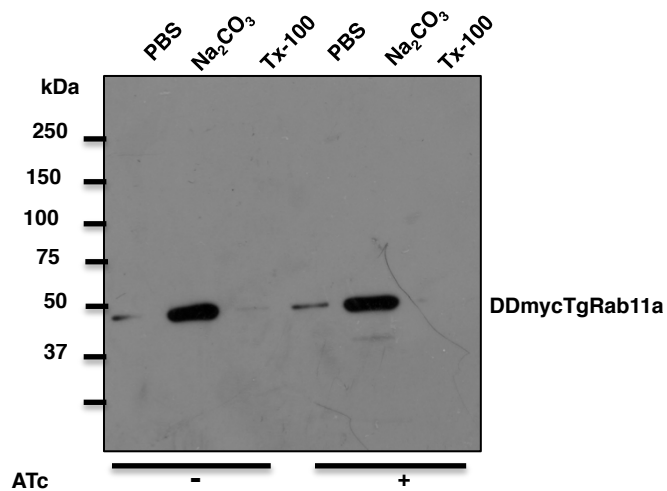**B**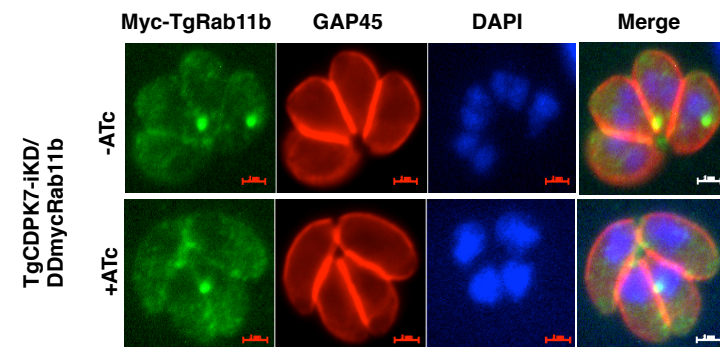**C**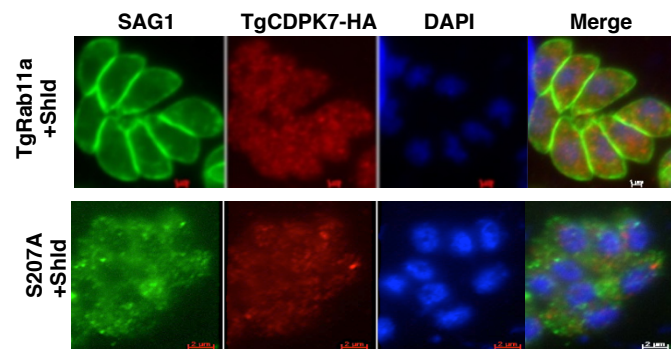

Supplement: S9 Fig — A. TgCDPK7-iKD/DDmycTgRab11a parasites were treated with ATc in the presence of Shld-1. Subsequently, parasite pellets were isolated and used for extracting proteins in PBS, Na2CO3 pH 11.0 or 0.5% Triton X-100. Western blotting using anti-myc antibody was performed on various fractions. B. TgCDPK7-iKD/DDmycTgRab11b in which TgRab11b was expressed in the presence of Shld-1 as N-terminal DD myc tag fusion protein in TgCDPK7-iKD background was treated with ATc followed by IFA using anti-myc antibody for detecting TgRab11b and anti-GAP45. C. TgCDPK7-HA/DDmycTgRab11a WT or S207A parasites were treated with Shld-1 for 4h. Subsequently, IFA was performed using anti-HA (to detect TgCDPK7-HA) and SAG1 antibodies. There was no significant difference in TgCDPK7 localization whereas SAG1 was mislocalized in TgRab11a S207A parasites as described in the main text. (PDF) [file ppat.1009325.s012.pdf]

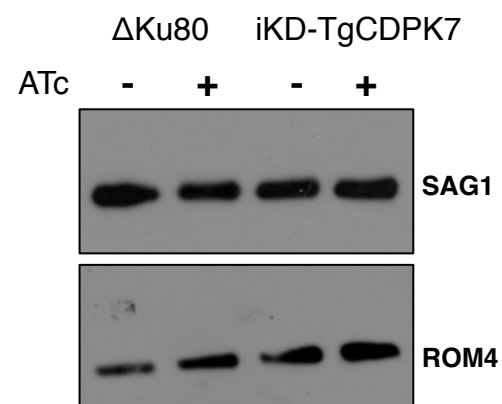

Supplement: S11 Fig — Western blotting using anti-SAG1/ROM4 antibody showed unaltered protein expression upon TgCDPK7 depletion with ATc for 72 hours. (PDF) [file ppat.1009325.s014.pdf]

A

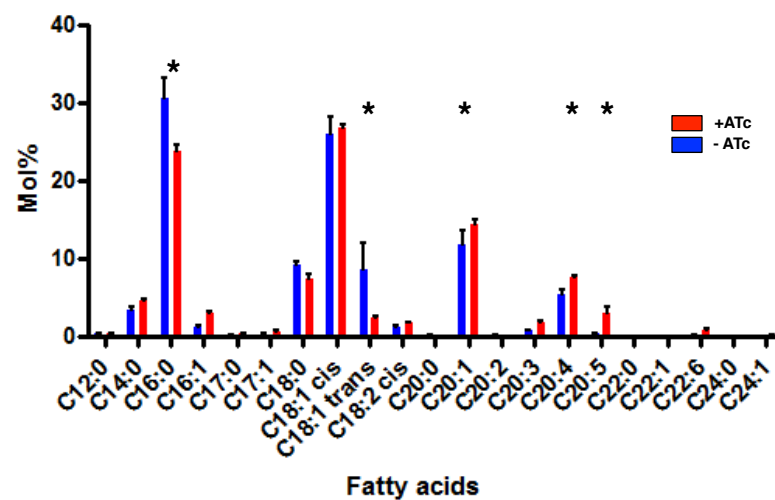

B

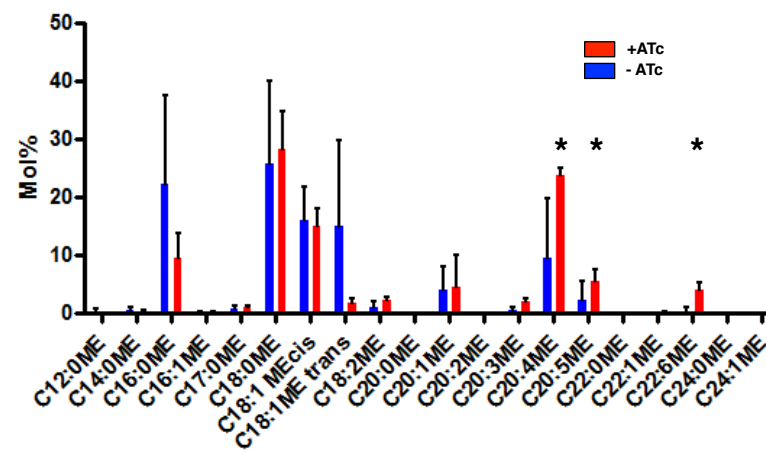

C

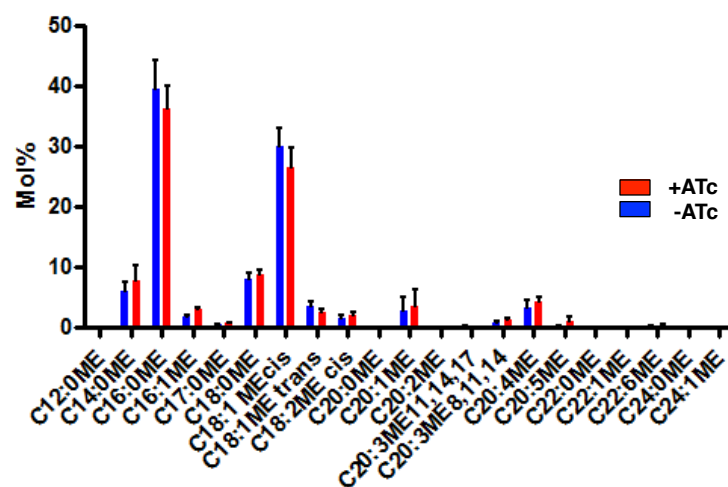

Supplement: S12 Fig — PLs were extracted from TgCDPK7-iKD tachyzoites grown in the presence or absence of ATc as described in Panel A. Relative abundance of individual molecular species in total FA (A), which shows the significant decrease of C16:0, C18:1 Trans and the significant increase in C20:1, C20:4 and C20:5 upon TgCDPK7 depletion. FA profiles were also quantified for the phospholipid classes PE (B) and PC (C) (mean±, SE, *n = 3, p<0.05, t-test (B,C)/ANOVA(A)). (PDF) [file ppat.1009325.s015.pdf]

A

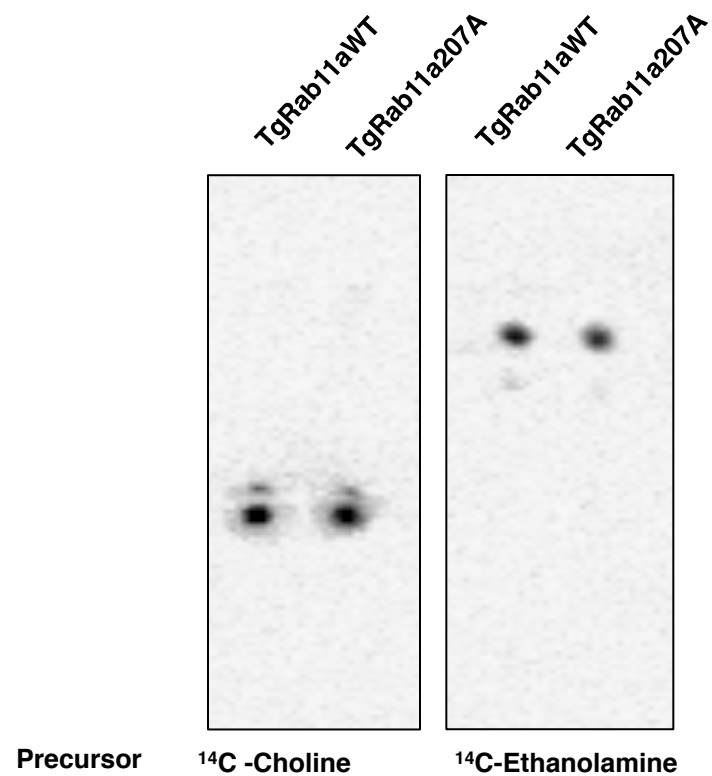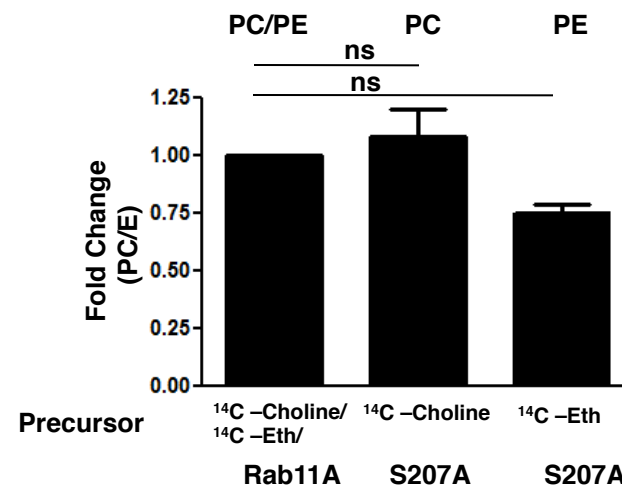

B

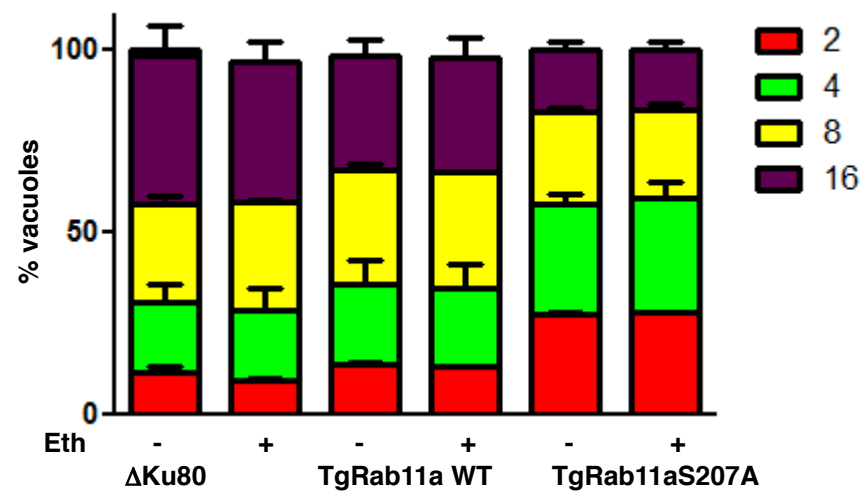

Supplement: S13 Fig — A. Metabolic labeling to monitor the synthesis of PC and PE via the parasite Kennedy pathway in parasites overexpressing either WT TgRab11a or its S207A mutant as described earlier in Fig 5B. Extracellular tachyzoites were incubated with 14C-Eth or 14C-Cho in the presence or absence of Shield-1 (16h treatment). Subsequently, lipids were extracted and radiolabeled lipids were detected by phosphorimaging of TLC. There was no significant difference in PC or PE levels. Right Panel, Densitometry of spots corresponding to PC/PE was performed and fold change in PC/PE in S207A mutant with respect to wild type TgRab11a was determined (Mean ± SE, ANOVA, n = 2, ns-not significant). B. WT TgRab11a or its S207A mutant parasites were incubated with or without 200 μM ethanolamine in the presence of Shield-1 for 16 hours. The number of parasites per vacuole was determined after 24h. Data represent Mean ± SE, n = 3 and at least 200 vacuoles were counted for each condition. (PDF) [file ppat.1009325.s016.pdf]

A

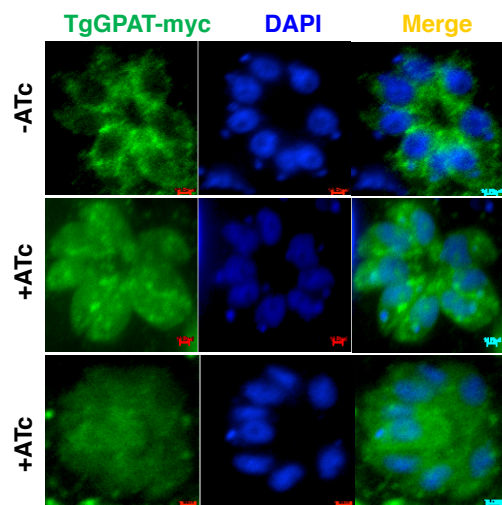

C

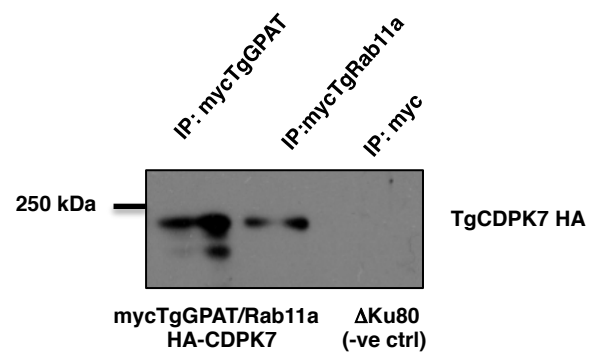

B

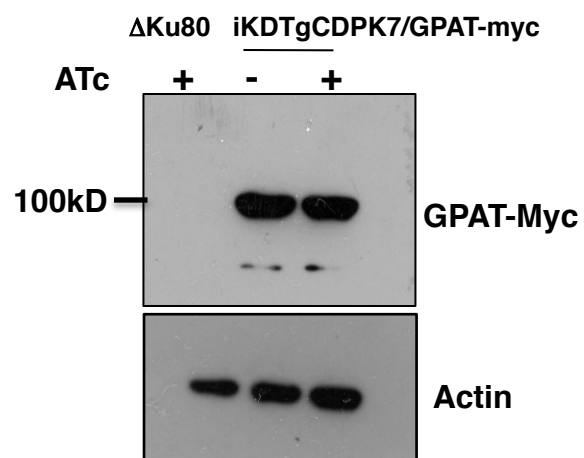

D

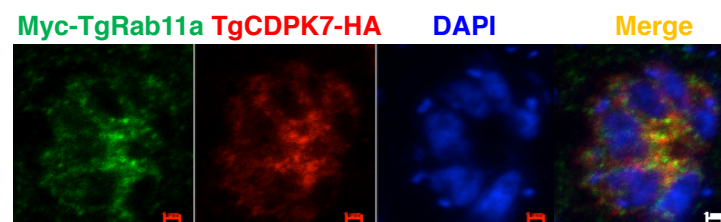

Supplement: S14 Fig — A. GPAT was myc-tagged at endogenous locus in its linker region as described in Fig 6A in TgCDPK7-iKD parasites (TgCDPK7-iKD/GPAT-myc). ATc was added to deplete TgCDPK7 followed by IFA for GPAT-myc as described in Fig 6D. There was a significant change in localization of GPAT from perinuclear ER like compartment (-ATc) to predominantly parasite cytoplasm upon TgCDPK7 depletion (+ATc). B. GPAT-myc/TgCDPK7-iKD parasites were treated with ATc for 72h followed by Western blotting with anti-myc antibody to detect GPAT-Myc. There was no significant change in GPAT expression upon ATc addition. C. TgCDPK7-HA/GPAT-Myc or TgCDPK7-HA/DDMyc-Rab11a parasites were cultured and parasite lysates were prepared from these or parental (ΔKu80) parasites and were used for IP with anti-myc. Subsequently, Western blotting was performed on GPAT-myc IP and DDmyc-Rab11a-IP using anti-HA antibody to detect TgCDPK7-HA. A band corresponding to TgCDPK7-HA was observed only in IP from transgenic parasites. No band was observed in the case of ΔKu80 line. D. TgCDPK7-HA/DDMyc-TgRab11a parasites were grown in presence of Shield-1 for 4h. IFA was performed using anti-myc and anti-HA antibodies. There were a few TgCDPK7 puncta that co-localized with TgRab11a-puncta. (PDF) [file ppat.1009325.s017.pdf]

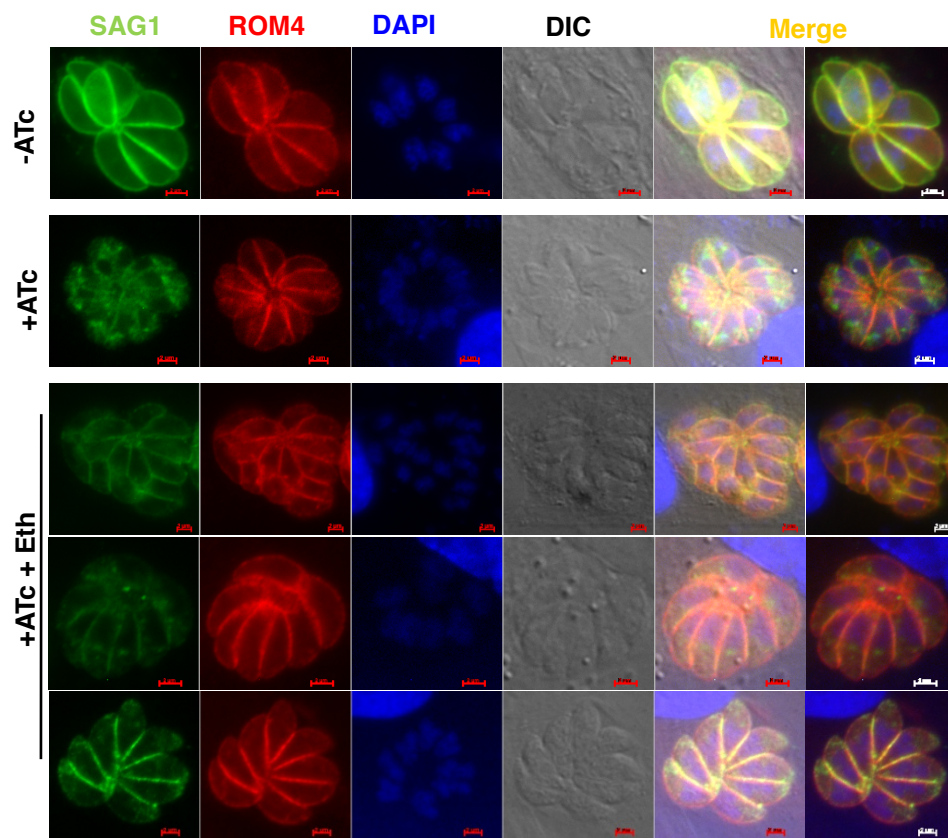

Supplement: S15 Fig — TgCDPK7-iKD parasites were preincubated for 48h with ATc and were subsequently allowed to invade fresh HFFs in the presence or absence of ATc for 24h. In one case, 200 μM of Eth was added for 120h to cultures prior to addition of ATc. Subsequently, IFA was performed for SAG1 and ROM4. The addition of Eth caused a partial restoration of pellicle staining of SAG1. SAG1 was present at pellicle as well as vesicles but the staining was reduced in comparison to -ATc cells. (PDF) [file ppat.1009325.s018.pdf]
