# Supplementary material for: Protein kinase TgCDPK7 regulates vesicular trafficking and phospholipid synthesis in Toxoplasma gondii
Source: PLoS Pathog. 2021 Feb 26;17(2):e1009325. doi: 10.1371/journal.ppat.1009325 (PMC7909640; doi:10.1371/journal.ppat.1009325)
Supplement: S10 Fig — Protein-protein interactions were predicted between differentially phosphorylated proteins in TgCDPK7-iKD proteins upon ATc addition (Fig 2B and S1 Data, 1.5) using STRING resource. The analysis exhibited high confidence protein-protein interactions between the candidate genes. The modules involving TgGPAT and TgRab11a discussed in Fig 2D and 2E are encircled. (PDF) [file ppat.1009325.s013.pdf]

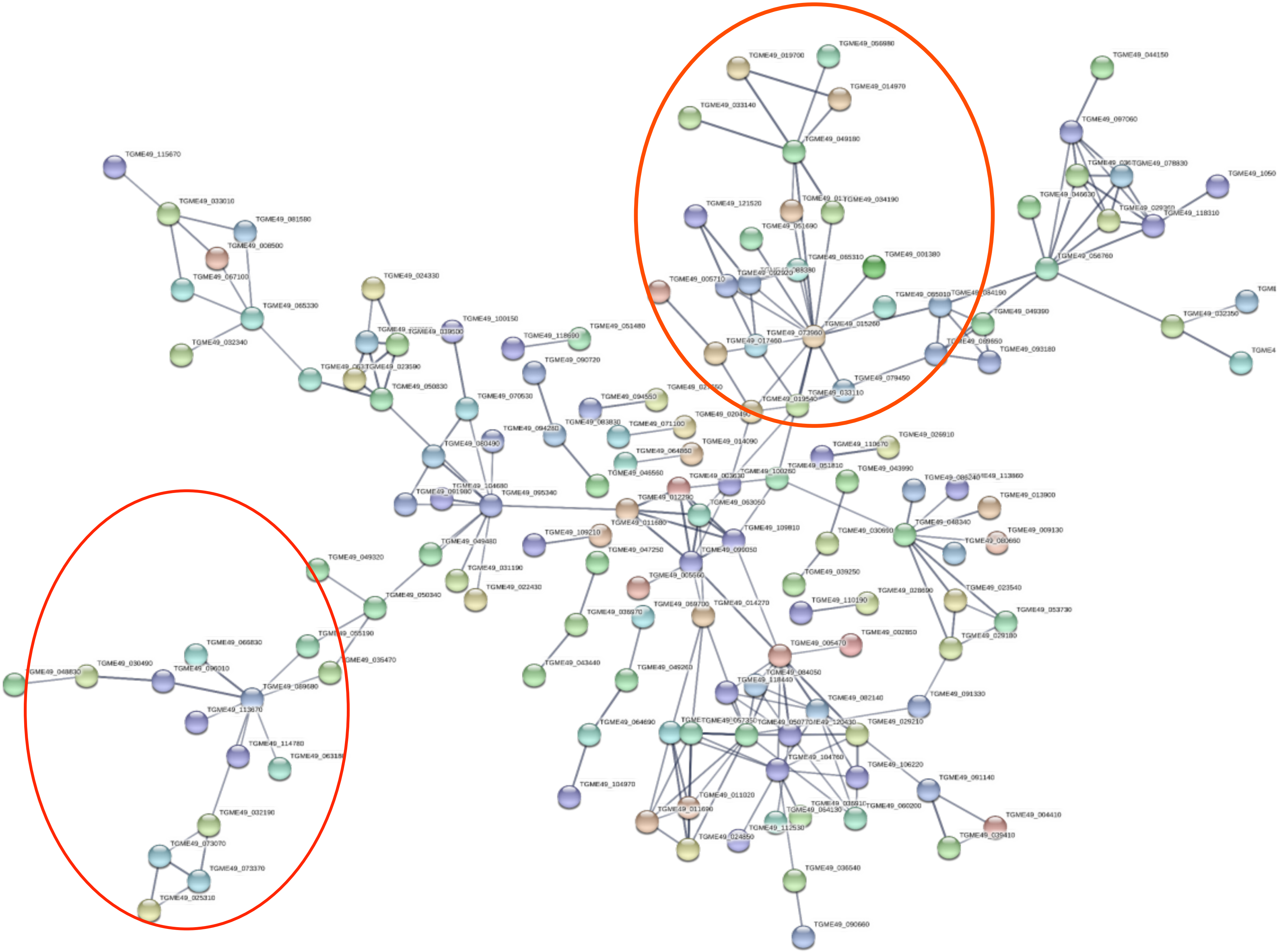

Supp. Fig. S10
